# Absolute abundance unveils *Basidiobolus* as a cross-domain bridge indirectly bolstering gut microbiome homeostasis

**DOI:** 10.1093/ismejo/wraf150

**Published:** 2025-07-21

**Authors:** Mitra Ghotbi, Jason E Stajich, Jason W Dallas, Alexander J Rurik, Chloe Cummins, Lluvia Vargas-Gastélum, Marjan Ghotbi, Joseph W Spatafora, Kian Kelly, Nicholas Reed Alexander, Kylie C Moe, Kimberly C Syring, Leila Shadmani, Julissa Perez-Marron, Donald M Walker

**Affiliations:** Department of Biology, Middle Tennessee State University, 1301 East Main Street, Murfreesboro, TN 37132, United States; Department of Plant Pathology & Microbiology, University of California Riverside, 900 University Avenue, Riverside, CA 92521, United States; Department of Biology, Middle Tennessee State University, 1301 East Main Street, Murfreesboro, TN 37132, United States; Department of Biology, Middle Tennessee State University, 1301 East Main Street, Murfreesboro, TN 37132, United States; Department of Biology, Middle Tennessee State University, 1301 East Main Street, Murfreesboro, TN 37132, United States; Department of Botany and Plant Pathology, Oregon State University, 2701 SW Campus Way, Corvallis, OR 97331, United States; Marine Ecology Research Division, GEOMAR Helmholtz Centre for Ocean Research at Kiel, Wischhofstraße 1–3, 24148 Kiel, Schleswig-Holstein, Germany; Department of Botany and Plant Pathology, Oregon State University, 2701 SW Campus Way, Corvallis, OR 97331, United States; Department of Plant Pathology & Microbiology, University of California Riverside, 900 University Avenue, Riverside, CA 92521, United States; Department of Biology, Middle Tennessee State University, 1301 East Main Street, Murfreesboro, TN 37132, United States; Department of Biology, Middle Tennessee State University, 1301 East Main Street, Murfreesboro, TN 37132, United States; Department of Botany and Plant Pathology, Oregon State University, 2701 SW Campus Way, Corvallis, OR 97331, United States; Department of Plant Pathology & Microbiology, University of California Riverside, 900 University Avenue, Riverside, CA 92521, United States; Department of Plant Pathology & Microbiology, University of California Riverside, 900 University Avenue, Riverside, CA 92521, United States; Department of Biology, Middle Tennessee State University, 1301 East Main Street, Murfreesboro, TN 37132, United States

**Keywords:** cross-domain networks, herpetofauna, host microbiome, internal standard, microbial interaction

## Abstract

The host microbiome is integral to metabolism, immune function, and pathogen resistance. Yet, reliance on relative abundance in microbiome studies introduces compositional biases that obscure ecological interpretation, while the absence of robust tools for absolute abundance quantification has limited biological discovery. Here, we apply absolute abundance profiling to uncover host-specific microbial patterns across herpetofauna orders that are masked in relative abundance data. Relative- and absolute abundance-derived bacterial and fungal microbiomes exhibit divergent profiles shaped by compositional bias and multifactorial effects. Absolute abundance identified key genera, *Lactococcus*, *Parabacteroides,* and *Cetobacterium* in salamanders, and *Basidiobolus* and *Mortierella* in lizards, turtles, snakes, and tortoises, that consistently emerged as core taxa, revealing host-associated patterns previously obscured by compositional constraints*.* In closely related *Desmognathus* species, where environmental and phylogenetic variation was minimized, absolute abundance enabled finer resolution of microbiome dynamics and significantly reduced false discovery rates. Absolute abundance-based network analyses further revealed distinct keystone taxa between the relative and absolute abundance datasets. Despite low redundancy, *Basidiobolus* exhibited high network betweenness, efficiency, and degree, suggesting its role as a key connector between microbial modules and a contributor to overall network robustness. This predicted structural role aligns with Burt’s structural hole theory, which suggests that nodes linking otherwise disconnected modules occupy influential network positions. These findings underscore the value of absolute abundance in resolving microbial dynamics and supporting meaningful interpretation of host-microbiome associations. This advance is made possible by DspikeIn, a flexible wet-lab and computational framework that enhances ecological resolution and cross-study comparability.

## Graphical Abstract

**Graphical Abstract:**
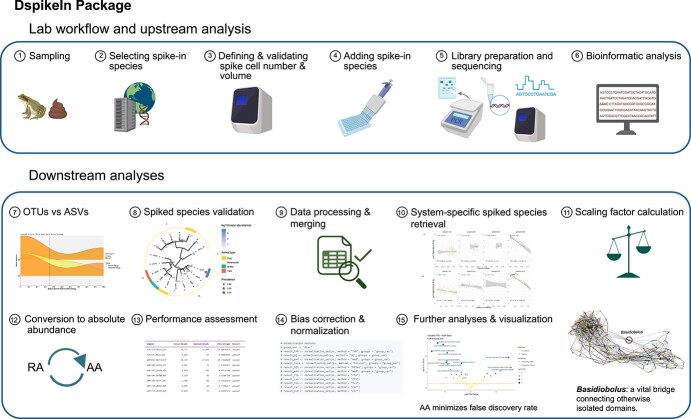

## Introduction

Growing interest in host microbiomes has underscored their vital role in host metabolism, immune function, and resilience to perturbations [1–3]. Central to these studies is the role of the core microbiome, a stable assemblage of microbial species consistently present across similar hosts or environments [4]. This foundational community offers a reference framework for defining a “healthy” microbiome [4, 5]. Complementing this concept, keystone taxa are species that, regardless of their abundance, can exert a prominent influence on ecosystem dynamics and functionality [6]. Defined by high connectivity, and centrality in microbial networks, keystone taxa are crucial for ecosystem stability, and their loss can trigger cascading effects or community reorganization [6, 7]. Among keystone taxa, *Basidiobolus* stands out as a filamentous fungus commonly found in the gastrointestinal tracts of reptiles and amphibians (herpetofauna) [8, 9]. Its unique ability to acquire genes via horizontal gene transfer from coexisting gut bacteria [8, 10] further highlights its ecological importance, making it an ideal model for studying bacterial-fungal interactions within herpetofauna gut microbiomes.

Host microbiome studies often employ high-throughput sequencing and multi-omics techniques to unravel complex microbial community dynamics and their interactions with host physiology [11–13]. These approaches have further enhanced the ability to conduct temporal and longitudinal studies, revealing how community shifts influence functional outcomes [14, 15]. However, the data generated through these methods is inherently compositional, reflecting the relative abundance (RA) of microbial communities [16, 17]. RA is constrained to a fixed total of one, meaning an increase in one taxon’s abundance proportionally reduces the apparent abundance of others. This compositional bias undermines the reliability of microbial community studies [18, 19], often introducing spurious negative correlations in association analyses and inflating false-positive rates in differential taxon analyses [19, 20], complicating the interpretation of microbial dynamics and interactions.

Analytical methods specifically developed to address compositional bias include data transformation techniques [21], and several applications including ALDEx2 [22], ANCOM-BC [18], DEICODE as a QIIME2 plug-in [23], Gneiss [24], Differential Ranking [25], and phylogenetic transform [26]. These methods employ taxon ratios to address compositional constraints, enhancing the accuracy and relevance of RA datasets [24, 27, 28]. Alongside computational advancements, laboratory techniques such as flow cytometry [29, 30], qPCR [28, 31], digital droplet PCR [28, 32], fluorescence *in situ* hybridization [12, 33], and fluorescence imaging with correlation spectroscopy [34] have been developed to estimate absolute abundance (AA) from relative counts. Although highly accurate, these techniques are often impractical for large-scale microbiome studies due to scalability limitations [16, 35]. As an alternative, the use of internal standards, or spike-ins, has emerged as a scalable approach for generating AA data from sequencing runs [16, 36, 37].

Spike-in approaches involve introducing a known external standard, such as synthetic DNA [35, 37] or non-native microbial whole cells, into a sample [36, 38]. This standard provides a reference point to accurately convert RA into AA values [16]. For whole-cell spike-ins, meticulous selection is essential to ensure that the introduced cells are entirely absent from the studied microbiomes [35, 37]. Whole-cell spike-in methods offer the dual advantage of providing AA and serving as reliable benchmarks to assess variability across key workflow stages, including DNA extraction and amplicon library preparation [16, 37]. Although their adoption is steadily gaining momentum, their application is currently limited to specific ecosystems, such as soil microbiomes [39, 40] and human gut microbiomes [36, 38]. There exists an untapped potential of these methods for broader applications, including the study of gut microbiomes in non-model organisms like herpetofauna. The alarming reality that 40.7% of amphibians and 21.1% of reptiles are threatened with extinction [41, 42] emphasizes the urgency of integrating microbiome research into conservation efforts [43–45]. Investigating herpetofauna microbiomes across diverse natural histories may uncover patterns of dysbiosis and its underlying causes [4, 46, 47], offering valuable insights to inform targeted strategies for mitigating biodiversity loss. Microbial interactions within host-associated communities are governed by complex networks that shape host physiology and health [38, 48, 49]. However, RA-based networks often distort ecological relationships by overlooking microbial population dynamics, inflating co-occurrence patterns, and obscuring inference of keystone taxa [30]. In contrast, AA-based networks provide a more accurate depiction of microbial dynamics and community structure [29], refining key properties such as connectivity, modularity, and centrality [30]. By offering a higher-resolution perspective on microbial interactions, AA-based approaches enhance our understanding of community dynamics. Despite growing recognition of the value of AA data for accurately characterizing microbiome dynamics, spanning host–microbe interactions [17, 28], microbial interactions [38], and heritability estimates [50], its integration has been hindered by the lack of a unified, bias-aware framework for converting RA into AA. To investigate the putative ecological role of *Basidiobolus* in herpetofauna gut microbiomes with improved quantitative accuracy, we developed DspikeIn: a modular wet-lab and R-based computational pipeline for robust AA estimation that addresses key limitations of RA-based approaches. Accordingly, this study tests three hypotheses: (a) spike-in recovery thresholds are system-specific rather than universally fixed; (b) RA-based analyses introduce compositional bias and inflate false discovery rates (FDR), distorting biological interpretation; and (c) AA-based analyses improve ecological resolution and reveal *Basidiobolus* as an abundant, central taxon contributing to microbial network robustness in herpetofauna gut ecosystems.

## Materials and methods

### Lab techniques and spike volume estimation

Detailed methods are provided in the Supplemental Information (Spike-in Volume Protocol). In brief, *Tetragenococcus halophilus* (bacterial spike; ATCC33315) and *Dekkera bruxellensis* (fungal spike; WLP4642) were selected for spiking gut microbiome samples, as they are absent from wildlife microbiome datasets (GenBank BioProjects: PRJNA1114724, PRJNA1114659). Stock cultures were grown for 72 hours in tryptic soy broth (*T. halophilus*) or potato dextrose broth (*D. bruxellensis*), diluted to OD600 values of 1.0, 0.1, 0.01, and 0.001, and DNA extracted with the Qiagen DNeasy Powersoil Pro Kit. These standards guided spike-in volumes to achieve 0.1–10% of total DNA [68]. To standardize input material, wood frog (*Lithobates sylvaticus*) fecal pellets (3.1 ± 1.6 mg) were processed as a 250 μl fecal slurry with and without spiked cells. Nine samples were analyzed to validate the protocol using two metrics: the expected increase in qPCR cycle threshold (Ct) values proportional to spiked cells and the corresponding increase in copy numbers for *T. halophilus* and *D. bruxellensis*. A synthetic DNA standard curve (16S-V4 rRNA for *T. halophilus* and ITS1 rDNA for *D. bruxellensis*) was used to convert Ct values into log copy numbers for statistical analysis. qPCR was used to compare synthetic DNA of *T. halophilus* and *D. bruxellensis* with DNA extracts and spiked wood frog fecal samples. SYBR Green qPCR assays (20 μl) used Quantabio SYBR Green Fastmix, primers, dsDNAse cleanup, and optimized DNA volumes (*D. bruxellensis*, 1 μl; *T. halophilus*, 3 μl) [51]. Primer pairs (515F/806R for bacteria, ITS1FI2/ITS2 for fungi [8]) were used for amplicon sequencing. Validation involved fecal samples spiked with *T. halophilus* (1874 copies) and *D. bruxellensis* (733 copies), DNA extraction, and MiSeq System (Illumina) sequencing.

### Bioinformatics

Demultiplexed reads were processed in QIIME2 (v2023.9) [23]. Barcodes and primers were removed using Cutadapt, and paired-end 16S rRNA reads were merged via FLASH (v1.2.11) and truncated to 225 bp (forward) and 220 bp (reverse). Two analysis pipelines were used: amplicon sequence variants (ASVs), generated via DADA2, and operational taxonomic units (OTUs), clustered de novo at 97% similarity. Taxonomy was assigned with the sklearn classifier using the SILVA 138 database (515F/806R region). ITS1 data followed a similar workflow, with ITSxpress [52] for region trimming and taxonomy assignment using the UNITE dynamic database (v10) [53].

#### Copy number correction

Gene copy number (GCN) correction was performed using the q2-gcn-norm plugin in QIIME2, referencing the rrnDB database (v5.7) [23], as GCN normalization is not integrated into the DspikeIn package. Alternatively, users may consider methods proposed by Louca et al. [54], such as PICRUSt, CopyRighter, and PAPRICA. Due to high variability in Internal Transcribed Spacer (ITS) gene copy numbers [55], GCN correction was not applied to ITS data; however, targeted adjustments may still help avoid overestimation of specific fungal taxa.

#### OTU versus ASV approach and phylogenetic validation of spiked species

OTU- and ASV-based approaches were evaluated using a spike-in standard to assess suitability for scaling RA to AA. Ten samples were used: two spiked blanks, two sterile swabs with spike-in species, and six spiked fecal samples. Both pipelines were processed identically, and spike-in recovery (%) was used to assess scaling accuracy. OTUs outperformed ASVs and were selected for downstream analysis. Spike-in OTUs were validated via phylogenetic comparison to a reference Sanger sequence using the validate_spikein_clade function in DspikeIn, and their distribution across samples was examined.

### DspikeIn cheat sheet

The DspikeIn package integrates with both Phyloseq and TreeSummarizedExperiment formats, enabling AA quantification across diverse microbiome studies. It supports single spike-in taxa or synthetic communities with customizable volumes and copy numbers. The package (full pipeline) features ten core functions, including: (i) spike-in validation, (ii) data preprocessing, (iii) system-specific spike recovery, (iv) scaling factor calculation, (v) absolute abundance conversion, (vi) bias correction and normalization, (vi) performance assessment, (viii) taxa exploration and filtering, (ix) network topology analysis, and (x) advanced visualization. Full documentation is available at (https://github.com/mghotbi/DspikeIn).

### System-specific spiked species recovery

The system-specific spiked species recovery threshold was determined by identifying meaningful shifts in key ecological characteristics of the microbial communities. This included assessing the point where spike-in integration (spiked reads per sample) began to affect native microbial metrics such as richness, evenness, beta dispersion, and structure [56]. Together, these metrics established a robust framework for optimal spiked species recovery.

### Relative versus absolute abundance comparisons

Comparative analyses of relative and absolute count datasets were conducted in R (version 4.3.2) using the DspikeIn, vegan [57], and phyloseq packages [58]. Alpha diversity indices (richness and evenness) were calculated directly from OTUs without rarefaction, which was deliberately avoided to preserve the integrity of diversity patterns and to identify the optimal system-specific spike-in range. Community structural deviation was quantified using Bray-Curtis distance-to-centroid measures, computed as each sample’s dissimilarity from the global community centroid. Subsequently, richness, evenness (Pielou’s and Hill number q = 1), distance-to-centroid, and total abundance (total reads) were regressed against spike-in read counts using the regression_plot function to determine the optimal spike-in range.

#### Core microbiome

To investigate whether converting to AA mitigates spurious inferences in group-level comparisons, we analyzed the core microbiome across hosts with varying natural histories (*n* = 312 samples from wild-caught salamanders, frogs, lizards, turtles, snakes, tortoises, toads, and crocodilians). Using the DspikeIn alluvial_plot function, we visualized co-abundant taxa (the cumulative top 70% of total abundance) across hosts and their natural histories. To minimize confounding effects arising from compositional biases and multifactorial ecological influences in RA vs. AA comparisons, we focused on three closely related host genera (*Desmognathus*, *Eurycea*, and *Plethodon*; n = 120 samples) within three adjacent Tennessee ecoregions. This targeted approach provided a balanced dataset, with results visualized using the taxa_barplot function.

#### Differential abundance and false discovery rate

Differential abundance was assessed using the perform_and_visualize_DA function with the DESeq2 method. Volcano and bar plots were used to compare FDR-adjusted *p*-values between RA and AA, highlighting taxa influencing microbial networks. DESeq2, which does not intrinsically account for compositionality, was used here, with spike-in standards applied to correct compositional bias. Methods like ALDEx2 [59] or ANCOM-BC [18] may offer more robust alternatives, particularly for datasets without spike-in correction. Presence/absence comparisons were used to evaluate taxonomic detection consistency between RA and AA OTU tables (Supplemental Information; Vignettes).

#### Microbial associations

Association networks can lose interpretability when environmental filtering effects become pronounced [60]. To minimize environmental variation and accurately capture taxon-taxon associations, we focused on two closely related salamander species, *Desmognathus imitator* and *Desmognathus monticola*, which share similar diets, native ranges, reproductive strategies, and habitat use. Filtered OTUs from both relative and absolute counts were used to construct association networks, enabling a comparative assessment of topological differences between RA- and AA-based networks. Bacterial and fungal OTUs were rarefied to ~4000 reads and merged for network analysis. Cross-domain microbial associations were inferred using the SpiecEasi package (v1.1.2) [61] and visualized in Cytoscape (v3.9.1). OTUs with >0.05% abundance and present in ≥40% of samples were retained. Network robustness was evaluated using the StARS method with 1000 subsamples (*rep.num* = 1000, *nlambda* = 300), selecting a lambda (~0.49) near the 0.5 stability threshold. Networks were inferred using SpiecEasi, which applies Meinshausen-Bühlmann neighborhood selection, sparse inverse covariance estimation, and centered log-ratio transformation to normalize taxonomic variation, reduce compositional bias, and correct for copy number effects [61]. *Basidiobolus-*centered subnetworks were extracted from the AA and RA networks to reveal taxon-specific association patterns. Network non-randomness was evaluated by testing: (i) degree distributions for power-law scaling using fit_power_law in igraph’ [62], and (ii) small-world topology via the Small-World Index (σ), compared to 1000 Erdős-Rényi random graphs [62, 63]; details in Supplementary Information. To test for shifts caused by the higher ITS copy number in fungi compared to the bacterial 16S rRNA marker, fungal abundances in the cross-domain network were scaled down by a factor of 10 [64].

### *Basidiobolus* role and network robustness tests

#### *Basidiobolus* first connections

Complete networks constructed from filtered OTUs served as the foundation for extracting nodes and association edges. Communities were identified using the Louvain algorithm for modularity optimization embedded in “igraph” [62]. *Basidiobolus* neighbors were identified using the extract_neighbors function in DspikeIn. First-order neighbors were directly connected nodes, and second-order neighbors were identified by iterating through them. Neighboring OTUs were grouped by class and visualized with “ggplot2” [65].

#### Network robustness

Modularity networks were constructed as described in Section 2.5.3, with nodes of the same color representing membership within the same module. Connectivity patterns classified nodes into four types based on within-module connectivity (*Zi*, which measures a node’s connectivity within its own module) and among-module connectivity (*Pi*, which quantifies its linkage to other modules): module hubs (highly connected within modules, Zi > 2.5, generalist); network hubs (highly connected across the entire network, Zi > 2.5 and Pi >0.62, super generalist); connectors (nodes linking different modules, Pi >0.62, generalist); and peripherals (nodes connected primarily within modules with few external connections, Zi < 2.5 and Pi <0.62, specialists) [66, 67].

Complete networks based on absolute counts capture the unaltered cross-domain microbial structure of the *D. monticola* and *D. imitator* gut microbiomes. Two additional networks were developed: the “network and module hubs removed” (NMHR) network, excluding critical nodes and their edges, and the “*Basidiobolus* subnetwork removed” (BSR) network, where nodes and edges associated with the *Basidiobolus* genus were removed. Negative edge weights, which hindered the calculation of network topological properties, were excluded. Network topological properties were compared using one-way ANOVA (ggpubr [68]), with results summarized in Table S1. [68].

Network robustness was further assessed by simulating random node removal and tracking changes in the largest connected component (LCC) using the simulate_network_robustness function in DspikeIn (*step* = 100), followed by regressing LCC size against the fraction of nodes removed. Constraint values quantified how concentrated a node’s connections were among its immediate neighbors, with high values indicating redundancy and low values suggesting potential “structural holes” where nodes bridge otherwise disconnected groups [69]. Redundancy was calculated as the sum of edges among a node’s neighbors, excluding direct links to the node itself [70]. Effective size was computed as node degree minus redundancy, while efficiency [69] was defined as the proportion of unique connections (effective size divided by degree). These metrics, along with betweenness centrality, were computed using the node_level_metrics function. To highlight and compare the structural roles of microbial taxa under targeted perturbations, we evaluated node-level metrics (efficiency, redundancy, and betweenness centrality) in relation to degree. Each metric was regressed against node degree to examine its association with network structure and potential contributions to robustness. Nodes falling within the top 40% for redundancy or efficiency were labeled and visualized using the ggplot2 package [65].

## Results

### Rationale for the selection of OTUs over ASVs and phylogenetic validation

OTU-based selection was driven by its ability to achieve 100% recovery efficiency of spiked species using De Novo Closed-Reference Clustering at a 97% similarity threshold in control samples. Compared to the ASV, the OTU approach also yielded a higher proportion of samples within the targeted recovery range for spiked species (Fig. S1A). Phylogenetic validation further confirmed the monophyletic grouping of spike-in OTUs and the clustering of sample reads with the reference sequences. The distribution of spike-in OTUs closely matched the expected proportions across all hosts, with the exception of the turtle samples (Fig. S1B-C).

### Range of spiked species recovery

**Figure 1:**
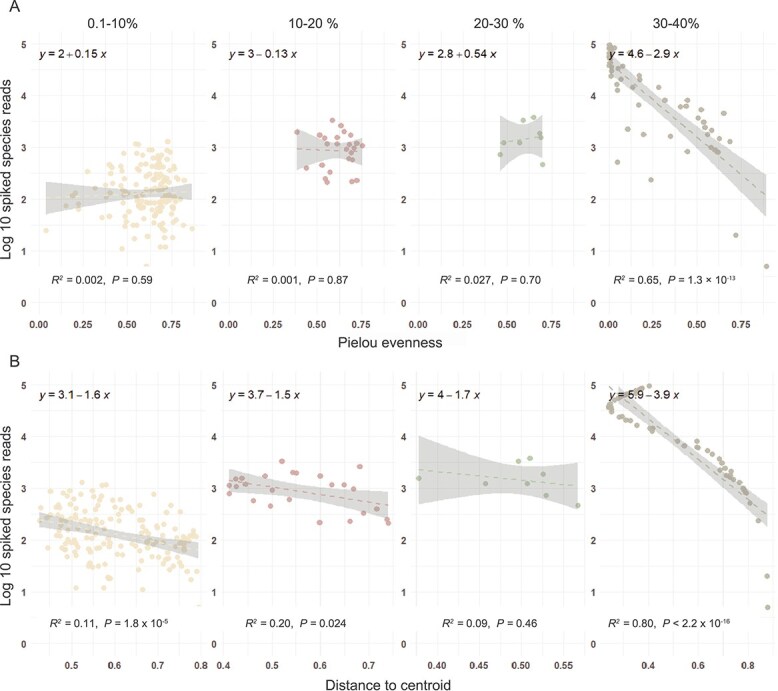
Linear relationship between log₁₀-transformed reads of the bacterial spike-in (*Tetragenococcus halophilus*) and two community metrics: (A) Pielou’s evenness (Hill-based evenness [q = 1] shown in Supplementary Fig. S2) and (B) distance to centroid, reflecting each sample’s dissimilarity from the global community centroid. Each panel represents a distinct spike-in recovery range: 0.1–10%, 10–20%, 20–30%, and 30–100%. Regression lines with corresponding R^2^ and p-values denote the strength and significance of associations within each interval. Shaded areas represent 95% confidence intervals. Fungal analogs are presented in Supplementary Fig. S3.

To determine the acceptable range of spiked species recovery, various biological metrics were regressed against spike-in read counts. In the bacterial dataset, evenness began to deviate beyond the 30% recovery range, which was further supported by rising *R*^2^ values in the regression analysis of Hill number q = 1, indicating a shift in community diversity (Figs. 1A-B and S2). Beyond 20%, distance-to-centroid metrics also showed increasing deviation, reflecting structural divergence from the native community. At recovery rates exceeding 20%, distance-to-centroid values became decoupled from spike-in read counts, while evenness continued to show a growing correlation. This suggests that spike-in recovery above 20% compromises the reliability of scaling factors and absolute abundance (AA) estimates. In the fungal (ITS) dataset, similar trends were observed. However, evenness remained relatively stable up to 40% spike-in recovery (Fig. S3A-D).

### Relative versus absolute abundance comparisons

#### Core microbiome dynamics

**Figure 2:**
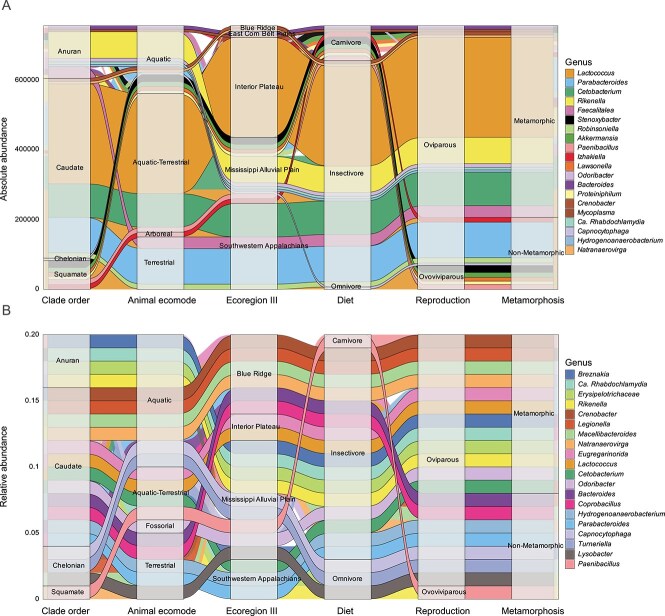
Distribution of core and prevalent bacterial genera, defined based on prevalence and abundance thresholds, across herpetofaunal orders, visualized using alluvial plots of (A) absolute abundance and (B) relative abundance. A consistent inclusion criterion was applied: genera with ≥3,000 reads in absolute abundance and ≥0.01% in relative abundance were retained. The alluvial plots illustrate the flow of these genera across host taxonomy (Anura and Caudata, i.e., frogs, toads, and salamanders) and natural history traits, including ecological mode, ecoregion, diet, reproductive strategy, and metamorphosis status. Similar color-code was used for genus in both plots for easy traceability. When genus-level classification was uninformative, the closest resolved taxonomic assignment was applied.

AA-based alluvial plots incorporating core microbiome detection revealed distinct distribution patterns of common and dominant taxa linked to host orders and natural history traits, such as diet, habitat, metamorphosis, and reproduction. In contrast, the RA core microbiome and mycobiome displayed distinct distribution patterns. Genera like *Lactococcus*, *Cetobacterium*, and *Parabacteroides* (bacteria), along with *Basidiobolus* and *Mortierella* (fungi), were more abundant across natural history traits in the AA plot, whereas RA plots showed genera more evenly distributed across traits and ecoregions (Figs. 2A-B and S4A-B).

Consistent detection of *Parabacteroides*, *Cetobacterium*, *Lactococcus*, *Rikenella*, and *Faecalitalea* across insectivorous hosts suggests a diet-associated core microbiome. *Lactococcus*, *Parabacteroides*, and *Cetobacterium* dominated the microbiomes of terrestrial, arboreal, and semi-aquatic hosts across Chelonian, Squamate, and Caudata orders. In contrast, the RA-based alluvial plot failed to reveal clear distribution patterns or consistent prevalence of these taxa across similar hosts, likely due to the compositional biases of RA data and the combined influence of multiple ecological factors.

**Figure 3:**
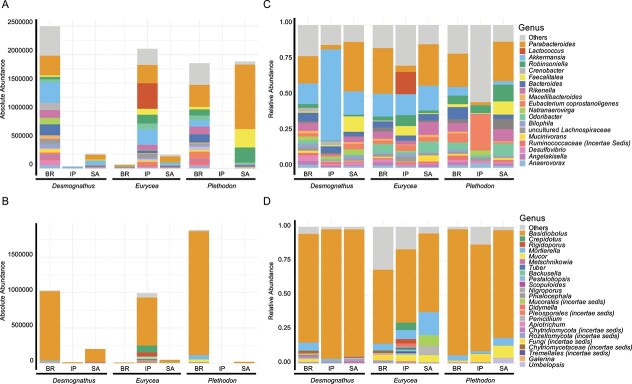
Variation in dominant gut bacteria (A and C) and fungi (B and D) among three genera (*Desmognathus*, *Eurycea*, and *Plethodon*) in the family *Plethodontidae* across three adjacent ecoregions (Southwestern Appalachians (SA), Interior Plateau (IP), Blue Ridge (BR)) in Tennessee, USA. Absolute abundance is shown in panels A and B, and relative abundance in C and D. Across these samples, absolute and relative abundance profiles showed 99.6% agreement in taxa detection based on presence/absence matrices. Taxa names beginning with “Ca” (e.g., Ca. *Rhabdochlamydia*) refer to *Candidatus* species.

To account for aggregate effects, the dataset was subsetted to three closely related host genera (*Desmognathus*, *Eurycea*, *Plethodon*) within three adjacent ecoregions, enabling accurate AA vs. RA comparisons. Bacterial and fungal community compositions differed between AA and RA across hosts and ecoregions (Fig. 3A-D). Despite these compositional shifts, presence/absence analysis showed strong agreement, with 99.6% of taxa consistently detected across both datasets (Supplementary Information). The gut microbiome of Blue Ridge *Desmognathus* was dominated by diverse taxa such as *Bacteroides, Rikenella, Bilophila*, *Akkermansia*, and *Parabacteroides*, while *Lactococcus* and *Akkermansia, and Parabacteroides* were primary contributors in *Eurycea* from the Interior Plateau. Unlike AA, RA profiling revealed consistent patterns, with *Odoribacter* and *Robinsoniella* more prevalent in both *Eurycea* and *Plethodon. Basidiobolus* emerged as the most abundant fungal genus across hosts based on absolute abundance (Fig. 3B), particularly in *Plethodon* and *Desmognathus*, with this pattern still evident, though less pronounced, in relative abundance (Fig. 3D).

#### Differential abundance and FDR

Volcano plots comparing differential abundance between two closely related *Desmognathus* species with similar natural histories demonstrated that AA yielded greater accuracy than RA by effectively eliminating the FDR in both the bacterial and fungal datasets (Fig. S5A-C). Moreover, plotting bacterial (Fig. S5A-B) and fungal enriched taxa across hosts further emphasized the limitations of RA in accurately capturing host-associated microbial abundance patterns (Fig. S6A-D). Differential abundance analyses showed that genera within *Firmicutes* (*Paenibacillus, Clostridium_sensu_stricto_13, Blautia, Mycoplasmopsis, Alicyclobacillus and, Bacteroidota*) were dominant in *D. monticola* based on AA, but appeared less prominent when using RA. In the fungal community, AA further revealed a higher prevalence of *Mucoromycota* genera in *D. monticola*, while *Ascomycota* and *Basidiomycota* genera were enriched in *D. imitator*.

#### Microbial cross-domain associations

**Figure 4:**
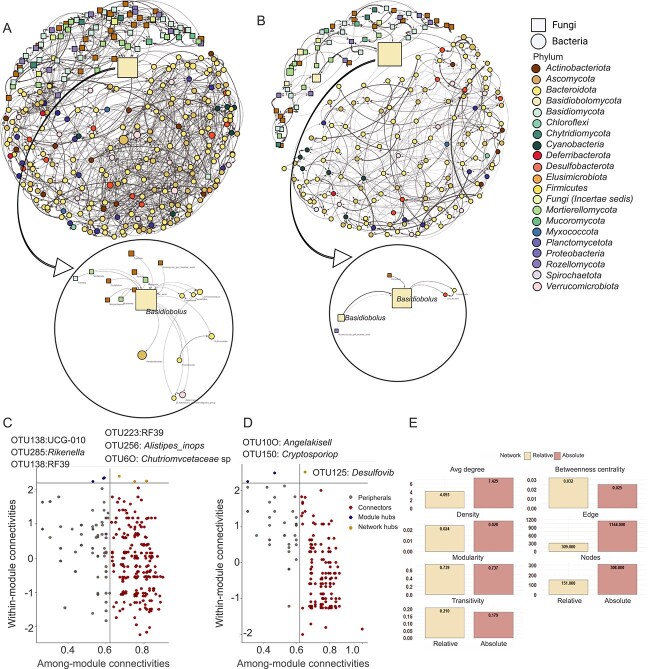
Cross-domain network associations and connectivity profiles of gut microbial communities from two salamander species (*Desmognathus monticola* and *Desmognathus imitator*) from the same ecoregion were constructed based on absolute (A) versus relative (B) abundance. OTUs were utilized for network construction. *Basidiobolus*-centered subnetworks were extracted from both network types to illustrate differences in taxon-level associations captured by AA versus RA data. Network nodes were categorized based on their connectivity in the network, including within-module and among-module connections, for absolute (C) and relative (D) counts. The nodes are color-coded to represent OTUs associated with specific phyla in the association network. Node size corresponds to the relativized abundance of each phylum. Node shapes indicate domains: Circles represent bacteria, and squares represent fungi. The edge colors depict the weight and sign of microbial associations: Gray edges represent positive associations. Mean topographic properties of relative count-based versus absolute count-based networks are also shown (E). Terminology can be found in Table S1.

Differences were observed in topological properties, including overall network structure (Fig. 4A-B). To illustrate this, we extracted *Basidiobolus*-centered subnetworks from both RA and AA networks. The AA subnetwork revealed a broader set of conditionally associated taxa, whereas the RA subnetwork showed fewer connections. Within- and among-module connectivity (Fig. 4C-D), along with node count (AA: 308, RA: 151), average degree (AA: 7.4, RA: 4.1), density (AA: 0.03, RA: 0.02), and edge number (AA: 1144, RA: 309), highlight key structural differences between networks (Fig. 4E). Network and module hubs differed between RA- and AA-based networks.

Both AA- and RA-based networks exhibited non-random topology, supported by heavy-tailed degree distributions (power-law fits: α = 6.65 and 16.76, respectively) and high Small-World Index values (σ = 231.88 for AA; σ = 159.14 for RA), reflecting clustered architectures. Full metrics are provided in Supplementary Table S2 and S3. Despite rarefying reads and using the Spiec-Easi package to mitigate GCN biases, we further adjusted fungal abundances by dividing them by 10 to account for fungal GCN being about an order of magnitude higher than bacterial GCN and the fungal spike-in *Dekkera*. This adjustment had minimal impact on network topology (Fig. S7A-C).

### *Basidiobolus* role and network robustness

#### *Basidiobolus* neighbors in network analysis

**Figure 5:**
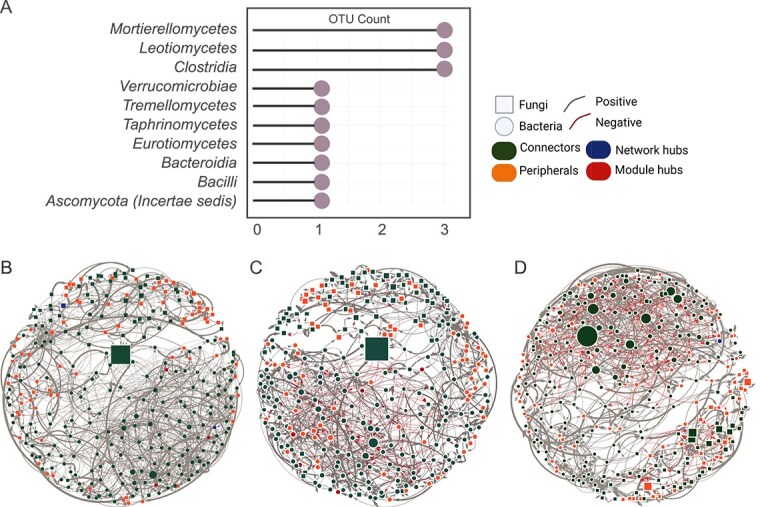
The distribution of OTUs by class for *Basidiobolus*’ first neighbors in the gut microbial networks of *D. monticola* and *D. imitator* is shown in (A), where the y-axis represents microbial classes, and the length of the lollipops reflects OTU proportions. Cross-domain network associations of *D. monticola* and *D. imitator* gut microbiomes using absolute counts without modification (complete) (B), after removing network and module hubs (C), after removing the *Basidiobolus* subnetwork (D) are shown. Networks are color-coded by connectivity patterns (see Fig. S8 for modularity networks). OTUs were utilized for network construction. Similar colors indicate taxa with comparable roles, node shapes differentiate bacteria (circles) from fungi (squares), and edge colors and thickness represent association weights, with gray indicating positive and red negative associations (terminology can be found in Table S1).

Complete networks and BSR comprised 18 modules, compared to 19 in NMHR (Fig. S8A-C). In the gut microbiome network, the first neighbors of *Basidiobolus* spanned diverse Classes, including *Mortierellomycetes*, *Leotiomycetes*, *Clostridia*, *Verrucomicrobiae*, *Tremellomycetes*, *Taphrinomycetes*, *Eurotiomycetes*, *Bacteroidia*, *Bacilli*, and *Ascomycota* unknown class (Fig. 5A). Five major classes dominated community members and second neighbors of *Basidiobolus*: *Dothideomycetes*, *Clostridia*, *Gammaproteobacteria*, *Bacteroidia*, and *Mortierellomycetes* (Fig. S8D-E).

#### Network topology and robustness

**Figure 6:**
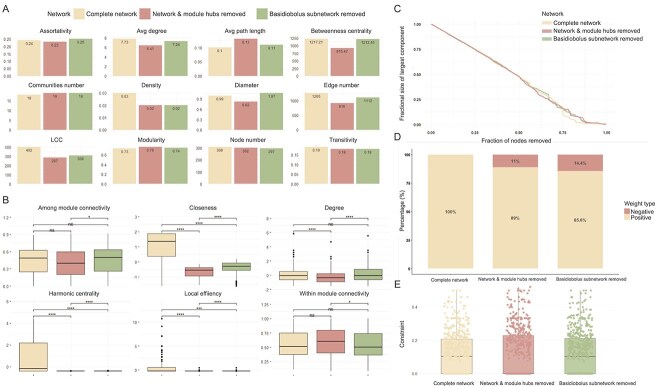
Comparison of average network topology metrics from the three cross-domain networks (refer to Fig. 5) (A). Node-based topology was analyzed using ANOVA (B), with significant pairwise differences indicated by asterisks (ns = non-significant; significance codes: *P* < .05 “^*^”, P < .001 “^***^”). Network robustness was evaluated by examining the effects of randomly removing nodes (C). Proportions of positive versus negative interactions across network types are shown (D), along with average constraint values (E). Network types are represented as follows: Complete network (cream), network with module hubs removed (dark pink), and *Basidiobolus* subnetwork removed (light green). Terminology is provided in Table S1.

To explore the ecological role of *Basidiobolus* in the herpetofauna gut microbiome, we analyzed three AA-based networks for *D. monticola* and *D. imitator*: the complete, NMHR, and BSR networks. Removing the *Basidiobolus* subnetwork had a greater impact on network shape and diameter than removal of network or module hubs, also reducing modularity and connectivity (Figs. 5B-D and S9A-C). Local efficiency, harmonic centrality, and closeness were similarly affected in both NMHR and BSR networks (Fig. 6A-E; full results in Fig. S10). The complete network featured only positive edges, while NMHR and BSR networks showed 11% and 14.4% more negative edges, respectively. Connectivity and modularity analyses underscored *Basidiobolus* as a key connector taxon facilitating cross-domain interactions (Figs. 5B-D and S8). The fractional size of the LCC decreased smoothly with the fraction of excluded nodes in both NMHR and BSR scenarios (Fig. 6C). Key robustness metrics, including LCC and modularity, showed minimal changes in the BSR network, with a moderate increase in average path length. High redundancy contributed to network resilience by buffering against disturbances through overlapping connections. In the complete network, species exhibited variable redundancy (mean = 1.2; median = 1.0). Nodes such as OTU256 and OTU268 occupied the top-right quadrant, acting as highly connected and locally stable hubs. Connectivity metrics, including betweenness and degree, identified *Basidiobolus* (OTU69) as a central hub and bridge (Fig. S11A-C). Positioned in a region of low redundancy but high betweenness, degree, and efficiency, *Basidiobolus* contributed little to conventional robustness yet served as a key connector across domains and communities.

## Discussion

### Absolute abundance enhances ecological resolution in host-microbiome systems

By mitigating compositional bias, AA enhanced ecological resolution, revealing microbial genera that were previously obscured or misrepresented in RA data. This clarity improved the detection of core microbiota, defined here as taxa consistently present across herpetofauna populations which likely contribute to essential ecological functions [5, 71]. These core members also exhibited stronger associations with host natural history traits, underscoring the utility of AA in resolving biologically meaningful patterns that RA may obscure (Figs. 2 and 3). Comparative network analyses between AA and RA revealed striking differences in topology and keystone membership, highlighting AA’s improved precision in identifying keystone taxa, defined as highly connected nodes that can shape microbiome structure and function beyond what their abundance suggests [72]. This improvement is consistent with findings from various quantitative studies across diverse hosts and habitats, particularly in host-associated systems [17, 29, 35, 36]. By correcting compositional bias, AA provides a clearer depiction of microbial communities and their ecological associations [30, 38], enabling more precise assessments of microbial dynamics, including associations with host phenotypes and environmental variables [28, 29, 38]. Ultimately, this advances our understanding of ecological and pathological relationships.

Using AA, we consistently identified *Lactococcus*, and *Cetobacterium* as dominant genera across herpetofauna taxa often obscured in relative abundance due to compositional distortion. Their consistent associations with host natural history traits and ecological adaptability underscore their role as core microbiome members and highlight the utility of AA for accurate biological interpretation. For instance, the prevalence of *Lactococcus* and *Cetobacterium* in herpetofauna gut microbiomes may reflect functional roles analogous to those observed in the giant Amazonian fish *(Arapaima gigas)* [73] and zebrafish (*Danio rerio*) [74]. In *A. gigas*, *Cetobacterium* constituted 55%-87% of the gut microbiome, playing key roles in host immune regulation and enhancing microbial community resilience, particularly against spring viremia of carp virus [73]. Similarly, in zebrafish, *Cetobacterium* demonstrated the capacity to synthesize vitamin B12, which stabilized the gut microbiome and significantly improved host resistance to *Aeromonas hydrophila* infection [74]. Likewise, *Lactococcus* species support gut diversity and probiotic function, as shown in swine where *Lactococcus lactis* enhanced growth, immune modulation, and microbiota composition [75]. In our study system, AA enhanced the resolution of core taxa enabling more precise investigation into their potential contributions to host physiology and ecological resilience. Although commonly associated with host function, such roles may vary across lineages and microbiome contexts.

### DspikeIn enables scalable AA for ecological interpretation

Given the limitations of RA data, we developed DspikeIn, a scalable workflow that integrates a biologically inert whole-cell spike-in with an R-based analytical pipeline to estimate AA. Validated in a non-model gut microbiome, the approach proved effective in contexts where conventional quantification methods often fall short. While qPCR is sensitive, it relies on precise standard curves [33, 76]; flow cytometry, though robust, is often cost-prohibitive and poorly scalable [29, 30]; and DNA-based spike-ins fail to account for upstream biases such as cell lysis and DNA extraction [35] (Table S4). In contrast, the DspikeIn workflow captures biases across the entire experimental pipeline, including DNA extraction, amplification, and sequencing, providing a biologically grounded benchmark for quantifying AA [36, 77–79]. Process-wide assessment and refinement of computational thresholds enabled by *DspikeIn* offer a reproducible, end-to-end framework that supports ecologically meaningful insights, revealing patterns and interactions often distorted by RA-based outputs [16, 33].

Debate continues regarding the use of OTUs versus ASVs, with contrasting perspectives emphasizing their respective advantages and limitations [80, 81]. Using whole-cell spike-ins, OTUs outperformed ASVs in converting RA to AA, achieving nearly 100% recovery of spiked species in positive controls. This may be due to intragenomic and intraspecific sequence variability [82], particularly in the ITS region, where clustering at a 97% similarity threshold reduces technical artifacts and prevents over-splitting of true biological variants [82, 83]. Studies using whole-cell spike-ins have favored OTUs for their reliability in generating scaling factors essential for accurate absolute abundance estimation [36, 37, 84, 85]. Since whole-cell spike-in studies rely on controlled abundance recovery, the inclusion of ASVs can introduce noise, making OTUs a more stable choice for our AA conversion [83]. While ASVs remain widely used for their fine-scale resolution [80, 86], a hybrid approach combining the strengths of both OTUs and ASVs could enhance AA-based microbiome studies. For ASV users, we recommend evaluating phylogenetic distances, comparing ASV relationships to a positive control (e.g., a Sanger FASTA file), and merging ASVs from spiked species for scaling factor computation, all of which can be efficiently performed using DspikeIn to ensure accuracy in absolute abundance estimation.

DspikeIn models the relationship between microbial metrics and spiked species reads, expanding the previously described 0.1–10% threshold range for spiked taxa [38]. This study demonstrated acceptable spiked species recovery thresholds of 20–30% for bacteria and 30–40% for fungi within the herpetofauna gut microbiome system. These thresholds reflect variation not solely due to marker type (e.g., 16S vs. ITS), but also host microbiome composition and upstream and downstream analyses. These findings emphasize that optimal spiked species recovery ranges are system-specific and should be tailored to the unique characteristics and metrics of each study. This post-assessment defines system-specific, community-level thresholds for acceptable spike-in recovery, recognizing that metrics such as evenness, richness, and distance to centroid are highly sensitive to perturbations [87]. While determining thresholds for the fungal and bacterial communities, we applied the lower bound in downstream analyses to ensure greater biological relevance.

### Inferred role of *Basidiobolus* in herpetofauna gut microbiome from AA-informed networks

We identified *Basidiobolus* as a core member and highly connected taxon within the gut microbiome networks of herpetofauna. This filamentous fungus, commonly found in the gastrointestinal tracts of diverse herpetofauna [88], likely contributes to the stability and functionality of their gut ecosystems [8]. *Basidiobolus* is not merely a central taxon but may serve as a cross-domain bridge that supports microbial stability within these networks. Its positive association with bacterial taxa, including *Bacteroides*, *Bacilli*, and *Verrucomicrobiae*, suggest that bacterial functions, such as polysaccharide digestion, short-chain fatty acid production, microbial balance, and mucosal integrity [89, 90], could be supported or stabilized through *Basidiobolus*-mediated interactions. Rather than functioning independently, these bacterial contributions likely operate within a fungal-scaffolded framework maintained by *Basidiobolus*.

Recent research highlights the critical role of the mycobiota in maintaining microbiota equilibrium and supporting gut health [91]. Microbial adaptability, such as siderophore production, is central to these interactions. Siderophores play a crucial role in bacterial iron acquisition under iron-limited conditions, promoting bacterial growth, shaping microbial community dynamics, and regulating virulence in pathogenic bacteria [92, 93]. Compounds like schizokinen and catecholates, produced by bacteria such as *Bacillus* spp., further promote microbial balance and host iron absorption [94–96]. These siderophore-mediated processes may be particularly relevant in the context of *Basidiobolus*’ associations with *Bacilli*, suggesting a possible fungal contribution to iron-mediated microbial cooperation.

Within the gut ecosystem, *Bacteroides* engages in both symbiotic and competitive interactions [97], exhibiting antigrowth and antivirulence activities against fungi [98]. This duality highlights the complexity of bacterial-fungal dynamics, where *Basidiobolus* may play a role in modulating these interactions. One hallmark of *Bacteroides* is its ability to degrade diverse polysaccharides through polysaccharide utilization loci, producing metabolites like short-chain fatty acids essential for gut health and homeostasis [89, 99]. Similarly, *Clostridium* genus contributes to gut function by producing beneficial metabolites such as butyrate [90, 100], which are essential for host metabolic and immune processes throughout the lifespan [100, 101].

Support for *Basidiobolus*’ potential role in regulating cross-domain microbial associations comes from network disassembly analysis, which revealed a measurable increase in predicted negative interactions (from 11.0% to 14.4%) following the removal of the *Basidiobolus* subnetwork. Although changes in negative edge density cannot be directly equated with microbiome homeostasis, the pattern suggests that *Basidiobolus* may play a role in sustaining microbial cohesion. Identifying such microbial connectors is essential for understanding biotic interactions that may influence secondary metabolite production, community resilience, and host-associated functions [102, 103]. *Basidiobolus* exhibited low redundancy but high centrality and network efficiency, suggesting a key role in supporting localized bacterial-fungal interactions (Fig. S11). Its contribution to microbial stability may extend beyond direct interactions, potentially shaping niche availability, metabolic flux, interspecies signaling, horizontal gene transfer [8, 10, 88], and bacterial dispersal via fungal highways [104, 105], though the latter remains unconfirmed. Network sensitivity to *Basidiobolus* removal further underscores its importance within these microbial communities. This architecture closely aligns with the concept of “structural holes” in network theory [69], where certain nodes preserve network cohesion by linking otherwise disconnected clusters. While *Basidiobolus* may not directly regulate microbiome balance, its network position suggests a potential role in maintaining microbial cohesion.

Microbial communities are shaped by a dynamic balance of positive and negative interactions, driven by diverse ecological processes [48, 106]. Positive associations, including cross-feeding, biofilm formation, and niche overlap, can promote microbial synergy and cohesion, while negative interactions, such as competition or amensalism, may destabilize community structure [48, 66, 102]. The interplay of these forces is central to microbiome robustness and resilience [49, 102]. To explore these dynamics, we used model-derived networks as a framework to infer potential biological mechanisms underpinning community structure. Within this context, a proposed framework [6], suggests that positive associations can promote stability but may also introduce vulnerability if key connectors, such as *Basidiobolus* in this system, are lost. This aligns with the stress-gradient hypothesis, where positive interactions provide temporary resilience under stress but increase fragility upon the loss of critical nodes [6, 107]. Diversity and redundancy further contribute to community resilience, as competitive interactions among well-adapted species help suppress overgrowth and stabilize network architecture [102, 108]. Robust systems often rely on numerous weaker interactions rather than a few dominant connections, enhancing adaptability under fluctuating conditions [49, 108]. *Basidiobolus* demonstrates flexibility in the gut environment, potentially facilitated by bacterial gene acquisition via horizontal transfer, as reported in anaerobic gut fungi [8, 10, 104, 109]. These network-inferred patterns position *Basidiobolus* as a compelling model for disentangling bacterial-fungal cross-domain dynamics in herpetofauna gut ecosystems [8]. Future experimental work will be critical to validate these predicted roles and to elucidate the mechanisms that underpin its proposed scaffold function.

## Conclusion

This study underscores the value of AA profiling in advancing ecological interpretation within microbiome research by mitigating compositional biases that are intrinsic to RA data. Despite a 99.6% taxonomic overlap between AA and RA profiles, AA analyses revealed substantial shifts in community structure driven by compositional distortion rather than true taxonomic turnover. By improving quantitative accuracy and reducing false discovery rates, AA profiling strengthened the associations between microbial community dynamics and host natural history. In our system, AA-informed network analyses identified *Basidiobolus* as a taxon with high centrality and cross-domain connectivity, features typically associated with structurally influential taxa. These properties may signal ecological importance, potentially shaped by horizontal gene transfer from coexisting bacteria such as *Bacilli*, or by cooperative interactions with key microbial groups including *Bacteroides*, *Clostridia*, and *Verrucomicrobiae*. Targeted removal of the *Basidiobolus* subnetwork, including its immediate connections, led to an increase in the proportion of predicted negative interactions, suggesting that *Basidiobolus* may help buffer the network against fragmentation. While these patterns are consistent with a potential mediating role in cross-domain interactions, they remain correlative and warrant functional validation through experimental approaches such as gnotobiotic models, metabolomics, or synthetic consortia. More broadly, our findings highlight the potential of AA-informed analyses to enhance the resolution of microbial interaction networks and to identify candidate keystone taxa that may be overlooked in RA-based studies. DspikeIn was developed as a workflow and tool to transform RA profiles into AA datasets, enabling reproducible and ecologically meaningful insights across diverse systems.

## Supplementary Material

Supplemental_R2_04072025_wraf150

Supplemental_Revised3_29072025_wraf150

## Data Availability

Data and DspikeIn package deposited on GitHub at https://github.com/mghotbi/DspikeIn. The raw sequence data are deposited in NCBI Sequence Read Archive under BioProject numbers PRJNA1202922, and PRJNA1210664.
