# Supplementary material for: Absolute abundance unveils Basidiobolus as a cross-domain bridge indirectly bolstering gut microbiome homeostasis
Source: ISME J. 2025 Jul 21;19(1):wraf150. doi: 10.1093/ismejo/wraf150 (PMC12400927; doi:10.1093/ismejo/wraf150)
Supplement: Supplemental_Revised3_29072025_wraf150 [file supplemental_revised3_29072025_wraf150.docx]

Absolute abundance unveils *Basidiobolus* as a cross-domain bridge indirectly bolstering gut microbiome homeostasis

Mitra Ghotbi[^1^](https://ops.straive.com/eProofingOUP/ExecuteProofHandler.ashx?ExecuteAction=ProofLoad&token=2uF4SjHmu/YpjqQdXMb4fA&ChapterOrArticleOrBook=Article&Redirect=yes&ProceedToProof=true#aff1)[^*^](https://ops.straive.com/eProofingOUP/ExecuteProofHandler.ashx?ExecuteAction=ProofLoad&token=2uF4SjHmu/YpjqQdXMb4fA&ChapterOrArticleOrBook=Article&Redirect=yes&ProceedToProof=true#cor1) [ID](https://orcid.org/0000-0001-9185-9993); Jason E. Stajich[^2^](https://ops.straive.com/eProofingOUP/ExecuteProofHandler.ashx?ExecuteAction=ProofLoad&token=2uF4SjHmu/YpjqQdXMb4fA&ChapterOrArticleOrBook=Article&Redirect=yes&ProceedToProof=true#aff2) [ID](https://orcid.org/0000-0002-7591-0020); Jason W. Dallas[^1^](https://ops.straive.com/eProofingOUP/ExecuteProofHandler.ashx?ExecuteAction=ProofLoad&token=2uF4SjHmu/YpjqQdXMb4fA&ChapterOrArticleOrBook=Article&Redirect=yes&ProceedToProof=true#aff1) [ID](https://orcid.org/0000-0002-3159-0985); Alexander J. Rurik[^1^](https://ops.straive.com/eProofingOUP/ExecuteProofHandler.ashx?ExecuteAction=ProofLoad&token=2uF4SjHmu/YpjqQdXMb4fA&ChapterOrArticleOrBook=Article&Redirect=yes&ProceedToProof=true#aff1); Chloe Cummins[^1^](https://ops.straive.com/eProofingOUP/ExecuteProofHandler.ashx?ExecuteAction=ProofLoad&token=2uF4SjHmu/YpjqQdXMb4fA&ChapterOrArticleOrBook=Article&Redirect=yes&ProceedToProof=true#aff1); Lluvia Vargas-Gastélum [^3^](https://ops.straive.com/eProofingOUP/ExecuteProofHandler.ashx?ExecuteAction=ProofLoad&token=2uF4SjHmu/YpjqQdXMb4fA&ChapterOrArticleOrBook=Article&Redirect=yes&ProceedToProof=true#aff3) [ID](https://orcid.org/0000-0003-4393-3145); Marjan Ghotbi^[4](https://ops.straive.com/eProofingOUP/ExecuteProofHandler.ashx?ExecuteAction=ProofLoad&token=2uF4SjHmu/YpjqQdXMb4fA&ChapterOrArticleOrBook=Article&Redirect=yes&ProceedToProof=true#aff4)^ [ID](https://orcid.org/0000-0001-9185-9993); Joseph W. Spatafora[^3^](https://ops.straive.com/eProofingOUP/ExecuteProofHandler.ashx?ExecuteAction=ProofLoad&token=2uF4SjHmu/YpjqQdXMb4fA&ChapterOrArticleOrBook=Article&Redirect=yes&ProceedToProof=true#aff3); Kian Kelly[^2^](https://ops.straive.com/eProofingOUP/ExecuteProofHandler.ashx?ExecuteAction=ProofLoad&token=2uF4SjHmu/YpjqQdXMb4fA&ChapterOrArticleOrBook=Article&Redirect=yes&ProceedToProof=true#aff2); Nicholas Reed Alexander[^1^](https://ops.straive.com/eProofingOUP/ExecuteProofHandler.ashx?ExecuteAction=ProofLoad&token=2uF4SjHmu/YpjqQdXMb4fA&ChapterOrArticleOrBook=Article&Redirect=yes&ProceedToProof=true#aff1) [ID](https://orcid.org/0009-0004-7271-5520); Kylie C. Moe[^1^](https://ops.straive.com/eProofingOUP/ExecuteProofHandler.ashx?ExecuteAction=ProofLoad&token=2uF4SjHmu/YpjqQdXMb4fA&ChapterOrArticleOrBook=Article&Redirect=yes&ProceedToProof=true#aff1); Kimberly C. Syring^[3](https://ops.straive.com/eProofingOUP/ExecuteProofHandler.ashx?ExecuteAction=ProofLoad&token=2uF4SjHmu/YpjqQdXMb4fA&ChapterOrArticleOrBook=Article&Redirect=yes&ProceedToProof=true#aff3)^; Leila Shadmani[^2^](https://ops.straive.com/eProofingOUP/ExecuteProofHandler.ashx?ExecuteAction=ProofLoad&token=2uF4SjHmu/YpjqQdXMb4fA&ChapterOrArticleOrBook=Article&Redirect=yes&ProceedToProof=true#aff2); Julissa Peres-Marron^[2](https://ops.straive.com/eProofingOUP/ExecuteProofHandler.ashx?ExecuteAction=ProofLoad&token=2uF4SjHmu/YpjqQdXMb4fA&ChapterOrArticleOrBook=Article&Redirect=yes&ProceedToProof=true#aff2)^; Donald M. Walker^[1](https://ops.straive.com/eProofingOUP/ExecuteProofHandler.ashx?ExecuteAction=ProofLoad&token=2uF4SjHmu/YpjqQdXMb4fA&ChapterOrArticleOrBook=Article&Redirect=yes&ProceedToProof=true#aff1)^[*](https://ops.straive.com/eProofingOUP/ExecuteProofHandler.ashx?ExecuteAction=ProofLoad&token=2uF4SjHmu/YpjqQdXMb4fA&ChapterOrArticleOrBook=Article&Redirect=yes&ProceedToProof=true#cor1) [ID](https://orcid.org/0000-0003-3119-8809)

^1^Department of Biology, Middle Tennessee State University, 1301 East Main Street, Murfreesboro, TN 37132, United States

^2^Department of Plant Pathology & Microbiology, University of California Riverside, 900 University Avenue, Riverside, CA 92521, United States

^3^Department of Botany and Plant Pathology, Oregon State University, 2701 SW Campus Way, Corvallis, OR 97331, United States

^4^GEOMAR Helmholtz Centre for Ocean Research at Kiel, Wischhofstraße 1–3, 24148 Kiel, Schleswig-Holstein, Germany

*Corresponding authors:

Dr. Mitra Ghotbi, Department of Biology, Middle Tennessee State

University at Murfreesboro, MTSU Box 0060, SCI 2141, TN, USA.

Primary Phone: (615) 898-5307

Cellphone: +1 313 707 6077,

[mitra.ghotbi@gmail.com](mailto:mitra.ghotbi@gmail.com)

ORCID: 0000-0001-9185-9993

Dr. Donald M. Walker, Department of Biology, Middle Tennessee State,

University at Murfreesboro, Box 60, Rm SCI 2044, TN, USA.

Donald.Walker@mtsu.edu

ORCID: 0000-0003-3119-8809

# Supplemental Information

# Spike-in volume Protocol,

The species *Tetragenococcus halophilus* (bacterial spike; ATCC33315) and *Dekkera bruxellensis* (fungal spike; WLP4642-White Labs) were selected as taxa to spike into gut microbiome samples as they were not found in an extensive collection of wildlife skin or gut microbiome samples. Stock cell suspensions of both microbes were grown in either static tryptic soy broth (*T. halophilus*) or potato dextrose broth (*D. bruxellensis*) for 72 hours then serially diluted and optical density (OD600) determined on a ClarioStar plate reader. Cell suspensions with an optical density of 1.0, 0.1, 0.01, 0.001 were DNA extracted using the Qiagen DNeasy Powersoil Pro Kit. These DNA isolations were used as standards to determine the proper spike in volume of cells to represent 0.1-10% of a sample [1] . Fecal pellets (3.1 ± 1.6 mg; range = 1 – 5.1 mg) from an ongoing live animal study using wood frogs (Lithobates sylvaticus) were used to standardize the input material for the development of this protocol. A total of (n=9) samples were used to validate the spike in protocol. Each fecal sample was homogenized in 1mL of sterile molecular grade water then 250uL of fecal slurry was DNA extracted as above with and without spiked cells.

Two approaches were used to evaluate the target spike-in of 0.1-10%, the range of effective spike-in percentage described in [1], (including 1) an expected increase of qPCR cycle threshold (Ct) value that is proportional to the amount of spiked cells and 2) the expected increase in copy number of *T. halophilus* and *D. bruxellensis* in spiked vs. unspiked samples. A standard curve was generated using a synthetic fragment of DNA for the 16S-V4 rRNA and ITS1 rDNA regions of *T. halophilus* and *D. bruxellensis*, respectively. The standard curve was used to convert Ct values into log copy number for statistical analyses (detailed approach in [2, 3]) using the formula y = -0.2426x + 10.584 for *T. halophilus* and y = -0.3071x + 10.349 for *D. bruxellensis*, where x is the average Ct for each unknown sample.

Quantitative PCR (qPCR) was used to compare known copy numbers from synthetic DNA sequences of *T. halophilus* and *D. bruxellensis* to DNA extractions of *T. halophilus* and *D. bruxellensis* independently, and wood frog fecal samples with and without spiked cells. SYBR qPCR assays were run at 20ul total volume including 10ul 2X Quantabio PerfeCTa SYBR Green Fastmix, 1ul of 10uM forward and reverse primers, 1ul of ArcticEnzymes dsDNAse master mix clean up kit, and either 1ul of DNA for *D. bruxellensis* or 3ul for *T. halophilus*. Different volumes of DNA were chosen for amplification of bacteria and fungi due to previous optimization of library preparation and sequencing steps [3]. The 515F [4] and 806R [5] primers were chosen to amplify bacteria and ITS1FI2 [6] and ITS2 for fungi, as these are the same primers used during amplicon library preparation and sequencing. Cycling conditions on an Agilent AriaMX consisted of 95 C for 3 mins followed by 40 cycles of 95 C for 15 sec, 60 C for 30 sec and 72 C for 30 sec. Following amplification, a melt curve was generated under the following conditions including 95 C for 30 sec, and a melt from 60 C to 90 C increasing in resolution of 0.5 C in increments of a 5 sec soak time. To validate the spike in protocol we selected two sets of fecal samples including 360 samples from a diverse species pool of frogs, lizards, salamanders, and snakes and a more targeted approach of 122 fecal samples from three genera of salamanders from the Plethodontidae. Fecal samples were not weighed in the field, rather, a complete fecal pellet was diluted in an equal volume of sterile water and standardized volume of fecal slurry (250µL) extracted for independent samples. A volume of 1ul *T. halophilus* (1874 copies) and 1ul *D. bruxellensis* (733 copies) were spiked into each fecal sample then DNA was extracted as above, libraries constructed, and amplicon sequenced on an Illumina MiSeq as in [7] .

# Summary of the DspikeIn core features:

## 1.Validation of spiked species:

DspikeIn is designed for microbiome data analysis, seamlessly integrating with phyloseq (for marker-gene microbiome data) and TreeSummarizedExperiment (TSE) (for hierarchical biological data, including microbiomes). DspikeIn facilitates the verification of OTUs/ASVs originating from spiked species through phylogenetic tree construction (e.g., Bootstrap_phy_tree_with_cophenetic), ensuring accuracy in downstream analyses. 2. Data preprocessing: The package prepares data for analysis by merging multiple OTUs/ASVs associated with spiked species (e.g., Pre_processing_species_list). 3. Scaling factor extraction: It extracts scaling factors from preprocessed data (e.g., calculate_list_average_scaling_factors), providing the foundation for accurate microbial quantification. 4. Conversion to absolute abundance: DspikeIn transforms RA data into absolute counts using established scaling factors (e.g., convert_to_absolute_counts). 5. Bias correction and normalization: The package offers systemic bias correction and dataset normalization through various methods (e.g., normalization_set(ps, method = "tss")). 6. System-specific spiked species retrieval: DspikeIn enables detection of optimal system-specific spiked species retrieval through visualization (regression_plot). 7. Performance assessment: It evaluates the success or failure rates of spiked data retrieval (e.g., calculate_spike_percentage_list and conclusion), helping to guide decisions for current and future sample spiking strategies. 8. Exploration and filtering of taxa: Beyond absolute abundance calculations, DspikeIn offers pathways to explore and filter core and dominant taxa. Features include core microbiome analysis, Random Forest-based selection, and prevalence assessments, all paired with intuitive visualization options to aid interpretation.

## 2. Bias correction and normalization methods:

Normalization methods validated in the literature [8–10] have been selected and modified from several references and packages, including EdgeR, DESeq2, and others. These bias correction methods include:

a) Trimmed Mean of M-values (TMM) Normalization: A scaling normalization method used for RNA-seq count data to account for compositional differences between libraries [10]. b) Cumulative Sum Scaling (CSS): This method assumes equivalent count distributions for low-abundance genes across samples up to a certain threshold. It scales the invariant segment of each sample’s count distribution and normalizes counts based on invariant counts across samples. c) Total Sum Scaling (TSS): Converts the feature table into relative abundance by dividing the total reads of each sample. TSS is commonly used for generating relative abundance data. Rarefying: Randomly removes reads from different samples until they all have the same predefined number of reads, ensuring equal library size. This method is considered a standard approach in microbial ecology. d) Centered Log-Ratio Transformation (CLR): Computes log-ratios relative to the geometric mean of all features, used to handle compositional data. e) Counts per Million (CPM): Derived from the LEfSe algorithm, this method is recommended for datasets with very low counts and is often used in differential abundance analysis. f) Relative Log Expression (RLE): Assumes that most features are not differentially expressed and uses relative abundances to calculate the normalization factor. g) RLE is commonly used in RNA-seq normalization. DESeq Normalization: Normalizes counts based on the assumption that most genes are not differentially expressed. DESeq is widely used in RNA-seq data analysis. h) PoissonSeq: A normalization method specifically designed for RNA-seq data, available in the PoissonSeq package. i) Quantile Normalization (QN): Adjusts the distribution of counts so that different samples have the same distribution, used in high-throughput data analysis.

## 3. Core microbiome dynamics

*Rikenella* was unique to the Anuran clade in the Mississippi Alluvial Plain, correlating with carnivorous hosts with metamorphic physiology. *Lactococcus* was widespread across diverse hosts and habitats, while *Erysipelatoclostridium* occurred in oviparous and insectivorous hosts.

*Eurycea* exhibited a more balanced fungal community structure, with notable contributions from *Mortierella* and *Backusella* based on AA. Host- and region-specific patterns in the abundance of *Basidiobolus* and *Mortierella* were evident in AA but obscured in RA analyses.

OUT vs ASV and GCN correction debate

Similar to the ongoing debate regarding the use of OTUs versus ASVs, where contrasting views highlight both advantages and limitations [11–14], there are likewise differing perspectives on the utility of gene copy number correction for the 16S rRNA marker. Proponents argue for its potential to improve accuracy [15–18], while critics emphasize its limitations and potential drawbacks [19, 20]. To address these challenges, several novel approaches and modifications have been introduced to enhance the precision of copy number correction [20, 21]. Despite these innovative approaches, intraspecific variability in the ITS region, and occasionally intragenomic variability, necessitates clustering ITS sequences to achieve species-level resolution in community analyses [22]. Consequently, we opted not to apply copy number correction for ITS data and chose clustering approaches over ASV due to this inherent variability.

Currently, GCN correction is not integrated into the DspikeIn package, it can be applied to RA data prior to conversion to absolute counts using tools such as the q2-gcn-norm plugin in QIIME2 (rrnDB v5.7) [17] or methods outlined by [19] including PICRUSt, CopyRighter, and PAPRICA. However, due to the inherent variability in rDNA gene copy numbers, GCN correction for ITS data is generally not recommended [22], except when applied to specific taxa with well-characterized loci to enable accurate normalization of abundance.

## 4. Network topology and robustness test

To investigate the ecological role of *Basidiobolus* in the herptile gut microbiome, we analyzed three absolute abundance-based networks for *D. monticola* and *D. imitator*: a complete network, one with network and module hubs removed, and one excluding the Basidiobolus subnetwork. The removal of Basidiobolus had a greater impact on the shape and diameter of network compared to the removal of network and module hubs (Fig. 5 D-F, S10 A-D, and S11 A-D). Metrics such as local efficiency, harmonic centrality, and closeness were similarly affected by the removal of either the Basidiobolus subnetwork or network and module hubs (Fig. 6 A-F). Interestingly, the complete network displayed exclusively positive interactions; however, the removal of hubs led to an 11% increase in negative interactions, while removing the Basidiobolus subnetwork resulted in a 14.4% increase. Connectivity and modularity networks emphasized the role of individual taxa in maintaining network integrity, with Basidiobolus specifically identified as a connector taxon facilitating inter-module interactions (Fig. 4 A, 5 D-F, and S10 A-C).

Key metrics of network robustness, including the largest connected component (LCC) and modularity, showed minimal changes after removing the Basidiobolus subnetwork, with a moderate shift in average path length (Fig. 6 A-I). The fraction size of the LCC versus the fraction of excluded nodes exhibited a similar smooth decreasing trend in both scenarios: removal of network and module hubs and removal of the *Basidiobolus* subnetwork (Fig. 6 D). High redundancy indicates a robust network, as overlapping connections, providing functional buffering. Removing low-redundancy species from the network caused a ~38% reduction in LCC size (from 308 to 191), highlighting their crucial role in maintaining network connectivity, a key factor in overall network stability. In the complete network, species displayed varying redundancy levels, with a mean of 1.2 and a median of 1.0, reflecting the network's robustness. Nodes such as OTU256:Alistipes inops and OTU268 occupied key positions in the top-right quadrant, signifying their roles as both highly connected and locally stable hubs. The removal of network and module hubs reduced connectivity, redistributing nodes to lower degree and redundancy regions. Plotting nodes based on connectivity metrics, such as betweenness and degree, revealed that *Basidiobolus* (OTU69) exhibited both high betweenness and degree, underscoring its role as a central hub and bridge within the network (Fig. 7 A-C). *Basidiobolus* (OTU69) was positioned in the low redundancy, high degree, and efficiency region, indicating it may play a less critical role in stabilizing the network but serves as a key bridge among communities. In the complete network, the spatial position of *Basidiobolus* (OTU69) on the betweenness versus degree plot highlighted its structural importance as a bridge or broker. Its high effective size underscores its unique and essential role within the network.

# 5. Taxonomic Detection Agreement Between RA and AA

We quantified taxonomic detection agreement between RA and AA by calculating the proportion of shared taxa presence across matched samples, defined as the number of taxa detected in both methods divided by the total number of taxa detected in either method.

This accurately describes:

Taxonomic detection consistency was assessed by calculating the proportion of shared presence calls between RA and AA. Specifically, presence/absence matrices were generated from each OTU table, and the overlap was computed as:

$$\left( Eq.1 \right)Percent Shared=\frac{\sum(taxa present in both RA and AA)}{\sum(taxa present in either RA or AA)}$$

This metric represents the fraction of taxon-by-sample combinations that were jointly detected by both methods out of all those detected by at least one method. It quantifies overall detection concordance between RA and AA profiles.

Presence/absence comparison (99.6% percent of shared taxa) indicated concordance in taxon detection between RA and AA profiles. Observed differences reflect abundance distortions rather than taxa being lost or gained between approaches.

# 6. Power-law scaling and Small-World Index Analysis

To evaluate whether network structure deviated from randomness, we assessed: (i) the degree distribution using fit_power_law in the *igraph* package to test for power-law scaling; and (ii) small-world topology using the Small-World Index (σ), calculated by comparing clustering coefficients and path lengths to those of 1,000 Erdős-Rényi random graphs of equivalent size and density.

We computed the Small-World Index (SWI; σ) following the quantitative framework established by [23]. The SWI is defined as the ratio between the normalized clustering coefficient and the normalized average shortest path length of the empirical network, relative to an equivalent Erdős-Rényi (ER) random graph with the same number of nodes and edges.

$$\left( Eq.2 \right) \sigma=\frac{Global clustering coefficient real/Global clustering coefficient random}{Avg Path real/Avg Path random}$$

Values of σ > 1 are indicative of small-world architecture, characterized by high local interconnectedness and short global separation, properties commonly observed in biologically organized systems [24]. Clustering and path length were calculated using the transitivity and mean_distance functions from the igraph package in R. For comparison, we generated 100 ER random graphs using the sample_gnm function, each preserving the empirical network’s size and density, and averaged their corresponding​ values.

This analysis was performed on the complete (unperturbed) network (using RA and AA-counts), as small-world topology is a global structural property and not intended for comparative analysis across experimentally modified or node-depleted networks.

# References

1. Rao C et al. Multi-kingdom ecological drivers of microbiota assembly in preterm infants. *Nature* 2021;**591**:633–638. https://doi.org/10.1038/s41586-021-03241-8

2. Romer AS et al. Host microbiome responses to the Snake Fungal Disease pathogen (Ophidiomyces ophidiicola) are driven by changes in microbial richness. *Sci Rep* 2022;**12**. https://doi.org/10.1038/s41598-022-07042-5

3. Walker DM et al. Variation in the Slimy Salamander (Plethodon spp.) Skin and Gut-Microbial Assemblages Is Explained by Geographic Distance and Host Affinity. *Microb Ecol* 2020;**79**:985–997. https://doi.org/10.1007/s00248-019-01456-x

4. Parada AE, Needham DM, Fuhrman JA. Every base matters: Assessing small subunit rRNA primers for marine microbiomes with mock communities, time series and global field samples. *Environ Microbiol* 2016;**18**:1403–1414. https://doi.org/10.1111/1462-2920.13023

5. Apprill A et al. Minor revision to V4 region SSU rRNA 806R gene primer greatly increases detection of SAR11 bacterioplankton. *Aquatic Microbial Ecology* 2015;**75**:129–137. https://doi.org/10.3354/ame01753

6. Schmidt PA et al. Illumina metabarcoding of a soil fungal community. *Soil Biol Biochem* 2013;**65**:128–132. https://doi.org/10.1016/j.soilbio.2013.05.014

7. Vargas-Gastélum L et al. Herptile gut microbiomes: a natural system to study multi-kingdom interactions between filamentous fungi and bacteria. *mSphere* 2024;**9**. https://doi.org/10.1128/msphere.00475-23

8. Bolstad BM et al. A comparison of normalization methods for high density oligonucleotide array data based on variance and bias. *BIOINFORMATICS* . 2003.

9. Abbas-Aghababazadeh F, Li Q, Fridley BL. Comparison of normalization approaches for gene expression studies completed with highthroughput sequencing. *PLoS One* 2018;**13**. https://doi.org/10.1371/journal.pone.0206312

10. Robinson MD, Oshlack A. A scaling normalization method for differential expression analysis of RNA-seq data. 2010.

11. Callahan BJ, McMurdie PJ, Holmes SP. Exact sequence variants should replace operational taxonomic units in marker-gene data analysis. *ISME Journal* 2017;**11**:2639–2643. https://doi.org/10.1038/ismej.2017.119

12. Callahan BJ et al. High-throughput amplicon sequencing of the full-length 16S rRNA gene with single-nucleotide resolution. *Nucleic Acids Res* 2019;**47**:E103. https://doi.org/10.1093/NAR/GKZ569

13. Caruso V et al. Performance of Microbiome Sequence Inference Methods in Environments with Varying Biomass. *mSystems* 2019;**4**. https://doi.org/10.1128/msystems.00163-18

14. Chiarello M et al. Ranking the biases: The choice of OTUs vs. ASVs in 16S rRNA amplicon data analysis has stronger effects on diversity measures than rarefaction and OTU identity threshold. *PLoS One* 2022;**17**. https://doi.org/10.1371/journal.pone.0264443

15. Pollock J et al. The Madness of Microbiome: Attempting To Find Consensus “Best Practice” for 16S Microbiome Studies. *Appl Environ Microbiol* 2018;**84**. https://doi.org/10.1128/AEM.02627-17

16. Starke R, Pylro VS, Morais DK. 16S rRNA Gene Copy Number Normalization Does Not Provide More Reliable Conclusions in Metataxonomic Surveys. *Microb Ecol* 2021;**81**:535–539. https://doi.org/10.1007/s00248-020-01586-7

17. Stoddard SF et al. rrnDB: Improved tools for interpreting rRNA gene abundance in bacteria and archaea and a new foundation for future development. *Nucleic Acids Res* 2015;**43**:D593–D598. https://doi.org/10.1093/nar/gku1201

18. Chiarello M et al. Ranking the biases: The choice of OTUs vs. ASVs in 16S rRNA amplicon data analysis has stronger effects on diversity measures than rarefaction and OTU identity threshold. *PLoS One* 2022;**17**. https://doi.org/10.1371/journal.pone.0264443

19. Louca S, Doebeli M, Parfrey LW. Correcting for 16S rRNA gene copy numbers in microbiome surveys remains an unsolved problem. *Microbiome* 2018;**6**. https://doi.org/10.1186/s40168-018-0420-9

20. Gao Y, Wu M. Accounting for 16S rRNA copy number prediction uncertainty and its implications in bacterial diversity analyses. *ISME Communications* 2023;**3**. https://doi.org/10.1038/s43705-023-00266-0

21. Perisin M et al. 16Stimator: Statistical estimation of ribosomal gene copy numbers from draft genome assemblies. *ISME Journal* 2016;**10**:1020–1024. https://doi.org/10.1038/ismej.2015.161

22. Kauserud H. ITS alchemy: On the use of ITS as a DNA marker in fungal ecology. *Fungal Ecol* 2023;**65**:101274. https://doi.org/10.1016/j.funeco.2023.101274

23. Humphries MD, Gurney K. Network ‘small-world-ness’: A quantitative method for determining canonical network equivalence. *PLoS One* 2008;**3**. https://doi.org/10.1371/journal.pone.0002051

24. Cram JA et al. Cross-depth analysis of marine bacterial networks suggests downward propagation of temporal changes. *ISME Journal* 2015;**9**:2573–2586. https://doi.org/10.1038/ismej.2015.76

25. Coyte KZ, Schluter J, Foster KR. The ecology of the microbiome: Networks, competition, and stability. *Science (1979)* 2015;**350**:663–666. https://doi.org/10.1126/science.aad2602

26. Coyte KZ, Rakoff-Nahoum S. Understanding Competition and Cooperation within the Mammalian Gut Microbiome. *Current Biology* . 2019. Cell Press, 2019. , **29**: R538–R544

27. Deng Y et al. Molecular ecological network analyses. 2012.

28. Ghotbi M et al. Management and rhizosphere microbial associations modulate genetic-driven nitrogen fate. *Agric Ecosyst Environ* 2025;**378**:109308. https://doi.org/10.1016/j.agee.2024.109308

29. Kajihara KT, Hynson NA. Networks as tools for defining emergent properties of microbiomes and their stability. *Microbiome* 2024;**12**:184. https://doi.org/10.1186/s40168-024-01868-z

30. Van Der Heijden MGA, Hartmann M. Networking in the Plant Microbiome. 2016. https://doi.org/10.1371/journal.pbio.1002378.t001

31. Taylor SC et al. The Ultimate qPCR Experiment: Producing Publication Quality, Reproducible Data the First Time. *Trends Biotechnol* 2019;**37**:761–774. https://doi.org/10.1016/j.tibtech.2018.12.002

32. Wang C et al. The quest for environmental analytical microbiology: absolute quantitative microbiome using cellular internal standards. *Microbiome* . 2025. 2025. , **13**: 26

33. Tkacz A, Hortala M, Poole PS. Absolute quantitation of microbiota abundance in environmental samples. *Microbiome* 2018;**6**. https://doi.org/10.1186/s40168-018-0491-7

34. Props R et al. Absolute quantification of microbial taxon abundances. *ISME Journal* 2017;**11**:584–587. https://doi.org/10.1038/ismej.2016.117

35. Vandeputte D et al. Quantitative microbiome profiling links gut community variation to microbial load. *Nature* 2017;**551**:507–511. https://doi.org/10.1038/nature24460

36. Harrison JG et al. The quest for absolute abundance: The use of internal standards for DNA‐based community ecology. *Mol Ecol Resour* 2021;**21**:30–43. https://doi.org/10.1111/1755-0998.13247

37. Zaramela LS et al. synDNA—a Synthetic DNA Spike-in Method for Absolute Quantification of Shotgun Metagenomic Sequencing. *mSystems* 2022;**7**. https://doi.org/10.1128/msystems.00447-22

38. Tourlousse DM et al. Synthetic spike-in standards for high-throughput 16S rRNA gene amplicon sequencing. *Nucleic Acids Res* 2017;**45**:e23. https://doi.org/10.1093/nar/gkw984

39. Wang X et al. Current applications of absolute bacterial quantification in microbiome studies and decision-making regarding different biological questions. *Microorganisms* 2021;**9**. https://doi.org/10.3390/microorganisms9091797

40. Morton JT et al. Establishing microbial composition measurement standards with reference frames. *Nat Commun* 2019;**10**. https://doi.org/10.1038/s41467-019-10656-5

41. Stämmler F et al. Adjusting microbiome profiles for differences in microbial load by spike-in bacteria. *Microbiome* 2016;**4**. https://doi.org/10.1186/s40168-016-0175-0

42. Ding Z et al. Detecting and quantifying Veillonella by real-time quantitative PCR and droplet digital PCR. *Appl Microbiol Biotechnol* 2024;**108**:1–13. https://doi.org/10.1007/s00253-023-12861-1

43. Gohl DM et al. Systematic improvement of amplicon marker gene methods for increased accuracy in microbiome studies. *Nat Biotechnol* 2016;**34**:942–949. https://doi.org/10.1038/nbt.3601

44. Gobert G et al. Droplet digital PCR improves absolute quantification of viable lactic acid bacteria in faecal samples. *J Microbiol Methods* 2018;**148**:64–73. https://doi.org/10.1016/j.mimet.2018.03.004

# Supplemental Tables and Figures

Table S1 Terminology of metrics used to explain gut microbial associations [25–30]

| **Term** | **Definition** |
| --- | --- |
| AmongModuleConnectivity | The degree to which nodes connect across different groups or modules within the network. |
| WithinModuleConnectivity | The extent to which nodes connect within the same group or module. |
| Average path length | The average path length is the mean of the shortest path lengths between all pairs of nodes in a network. |
| Betweenness Centrality | A measure of how often a node lies on the shortest path between other nodes. |
| Closeness Centrality | Describes how quickly a node can interact with all other nodes. |
| Degree | The number of direct connections (edges) a node has to other nodes. |
| Edge Density | The proportion of possible edges that exist in the network |
| Global Efficiency | The average inverse shortest path length across all node pairs in the network. |
| Local Efficiency | Efficiency within the neighborhood of a node, reflecting the fault tolerance of the network at a local level. |
| Module Hubs | A group of nodes that are more interconnected with each other than with nodes outside the group. |
| Network Hubs | Key nodes that have a disproportionately high number of connections to other nodes. |
| Subnetwork | A smaller network that is part of a larger one, focusing on a specific subset of nodes and their connections. |
| Eigenvector Centrality | Measures the influence of a node based not just on its direct connections but also on the importance of the nodes it connects to. |
| Harmonic Centrality | A variation of closeness centrality that accounts for the distance of each node from others, favoring nodes that are closer to more nodes. |
| Coreness | Indicates how central a node is in the network |
| Authority Score | A measure of how important a node is within the network, based on the number of connections from important nodes pointing to it. |
| Transitivity | Quantifies how well nodes in a network cluster together. |
| Redundancy Ratio | The proportion of duplicated connections. Can be measured the degree of overlap in connections between nodes |
| Clustering Coefficient | Measures the degree to which nodes in a graph tend to cluster together. |
| Largest Connected Component | The size of the largest connected subgraph. |
| Constraint | Measures how much a node is constrained by its neighbors, often due to redundancy in connections |

Table S2 Summary of discrete power-law fit parameters for node degree distributions of the AA- and RA-based fungal co-occurrence networks. The exponent (α), lower bound (xmin), and Kolmogorov-Smirnov statistic (KS) are reported. Both networks show heavy-tailed degree distributions indicative of non-random structure, with the AA-based network displaying moderate hub structure and the RA-based network exhibiting a steeper decay.

| Network Type | Power-law Exponent (α) | xmin | KS Statistic |
| --- | --- | --- | --- |
| AA-based | 6.65 | 12 | 0.049 |
| RA-based | 16.76 | 9 | 0.044 |

Table S3 Comparison of small-world network properties for AA-based and RA-based networks. Values shown are the global clustering coefficient and average shortest path length calculated for each real network and its corresponding Erdős-Rényi (random) graph of equal size and density. The Small-World Index (SWI; σ) was calculated using $Equ.1$

|  | Mean clustering coefficient | | Average path length | | SWI (σ) |
| --- | --- | --- | --- | --- | --- |
| Network Type | Real | Random | Real | Random |  |
| AA-based | 0.179 | 0.021 | 0.098 | 2.637 | 231.88 |
| RA-based | 0.210 | 0.00234 | 0.180 | 0.319 | 159.14 |

Both AA- and RA-based networks exhibited non-random topology, supported by heavy-tailed degree distributions consistent with discrete power-law fits (AA: α = 6.65, KS = 0.049; RA: α = 16.76, KS = 0.044). The AA network showed moderate hub structure, while the RA network had a steeper decay, reflecting weaker hub dominance. Small-world analysis further confirmed non-random architecture, with high Small-World Index values (σ = 231.88 for AA; σ = 159.14 for RA), driven by substantially higher clustering and shorter path lengths than expected in random networks.

Table S4 Comparisons of various methods for quantifying absolute abundance, including their advantages, limitations, applications, and scalability

| Method | Principle | Advantages | Limitations | Applications | Output | Scalability | Accuracy | Computational Requirements |
| --- | --- | --- | --- | --- | --- | --- | --- | --- |
| qPCR-based Approaches [31–33] | - Uses standard curves or reference genes to quantify absolute abundance of DNA targets | - High sensitivity and specificity  - Can detect low-abundance targets  - Relatively fast and cost-effective  - Compatible with low biomass samples | - Requires accurate standard curves  - Prone to PCR inhibitors  - Possibility of primers mismatch  - Does not distinguish between live and dead cells  16S rRNA copy number calibration may be needed  - Does not provide information on the environmental in situ gene abundance | - Microbial community analysis, absolute quantitation, pathogen detection, gene expression studies | - Copies of target gene per unit volume or per sample | - Moderate: Can process multiple samples in parallel but limited by qPCR throughput | - High, but depends on standard curve precision (amplification efficiency and primer specificity can alter the results) | - Low: Requires basic qPCR data processing |
| Flow Cytometry [34–36] | - Measures fluorescence from labeled cells or particles in a fluid stream | - High-throughput  - Can distinguish live vs. dead cells, - Multiparametric analysis possible (allowing in-depth phenotypic profiling) | - Requires fluorescent labeling  - High instrument cost  - Need trained personnel - May not work well for heterogeneous samples  - Exclusion of background signals may be necessary  - Often requiring careful gating strategies or sample dilution.  - Lacks scalability for large sample sets | - Microbial quantification, immune cell profiling, single-cell analysis | - Cells per unit volume (e.g., cells/mL) | - High: Can analyze thousands of cells per second | - High, but depends on gating strategies and fluorescence markers | - Moderate: Requires data processing for gating and population analysis |
| Synthetic DNA Spike-in [37, 38] | - Known quantities of synthetic DNA added as an internal control for normalization in sequencing or qPCR | - Allows accurate normalization  - Can correct for PCR and sequencing biases  - Cost-effective for nucleic acid-based assays | - Cannot differentiate live vs. dead cells  - Does not correct for biases in DNA extraction and cell lysis  - Requires proper design to avoid cross-contamination | - Metagenomics, transcriptomics, microbial load quantification | - Ratio of target DNA to spike-in DNA | - High: Easily scalable with sequencing or qPCR workflows | -Moderate to High, depending on spike-in design and normalization | - High: Requires computational correction and normalization in sequencing data |
| Whole-Cell Spike-in [36, 39–41] | - Addition of known quantities of whole cells from a microbial species absent in the host microbiome as an external reference standard | - Allows accurate normalization  - Captures biases in DNA extraction, PCR, and sequencing  - Reflects real biological conditions better than synthetic DNA  - Suitable for absolute quantification of microbial cells, gene abundance, expression, and proteomics | - Requires culturable reference organisms  - Potential differences in lysis efficiency  - Complex standardization | - Microbial community analysis**,** fine-level microbiome studies, Metagenomics, gene expression studies (qPCR, RNA-Seq), proteomics | - Cells per unit volume, protein concentration, or gene expression fold change | - Moderate: Limited by the ability to culture and standardize spike-in organisms | - High: Depends on the standardization of spike-in cells | - Moderate to high: Requires normalization in sequencing, correction for technical noise, and normalization in qPCR, or proteomics data |
| Droplet Digital PCR (ddPCR)[39, 42–44] | - Partitioning of PCR reactions into thousands of nanoliter-sized droplets, each acting as an individual reaction, followed by end-point fluorescence detection | - Absolute quantification without standard curves - High precision and sensitivity - Tolerant to PCR inhibitors - Robust for low-copy targets | - Requires specialized (expensive) equipment - Lower throughput than qPCR - Cannot differentiate live/dead cells - Limited multiplexing  - Dilutions are required for high concentrated template  - may require a large number of replicates | - Microbial quantification, rare target detection, low-biomass microbiome samples, pathogen diagnostics | - Copies per µL of sample | - Moderate: Parallel wells possible, but droplet generation slows throughput | - High: High reproducibility, especially for low-abundance targets | - Moderate: Even though ddPCR gives absolute copy numbers (e.g., copies per µL), normalization may still be necessary |

Figure S1 Panel (A) evaluates the accuracy of ASV and Operational Taxonomic Unit (OTU) approaches in retrieving spiked species, with retrieval quantified using the calculate_spike_percentage function in DspikeIn. Panel (B) compares the phylogenetic distances between reference and OTUs rooted in the spiked species present in the samples. Panel (C) shows the distribution and prevalence of spiked species OTUs across animal type using plot_spikein_tree_diagnostic function. Right color bar indicates the log10 of mean abundance of each OTU. Stars represent the prevalence of each OTU in the samples. OTUs with larger stars are more consistently detected across samples within the respective animal type.


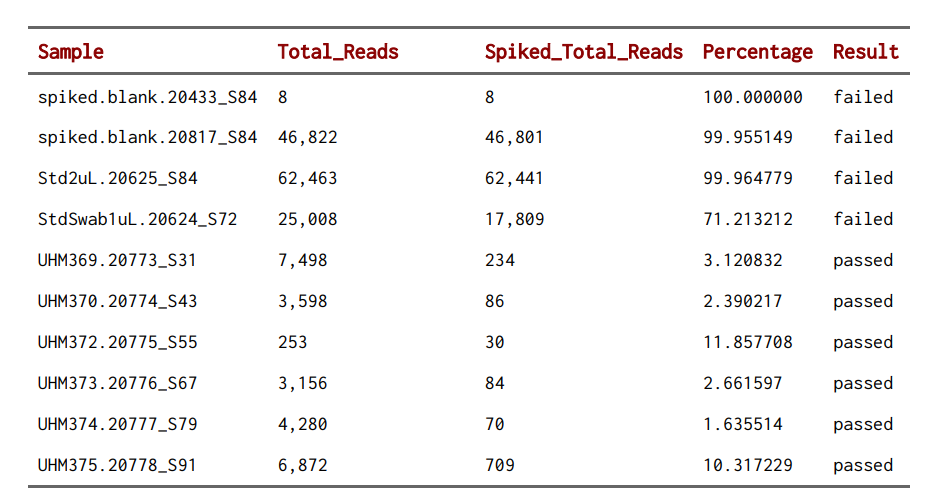


C

A

B


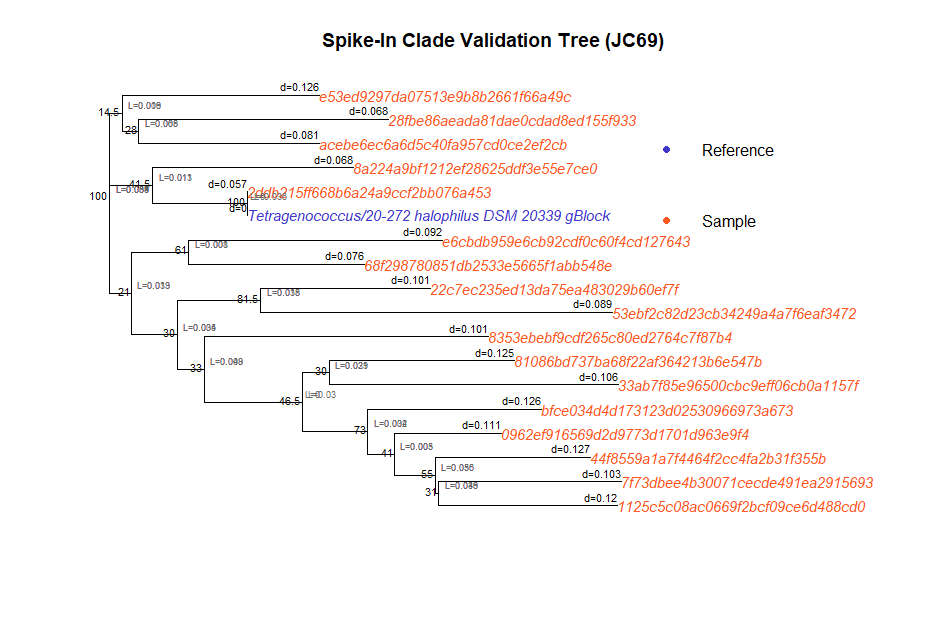

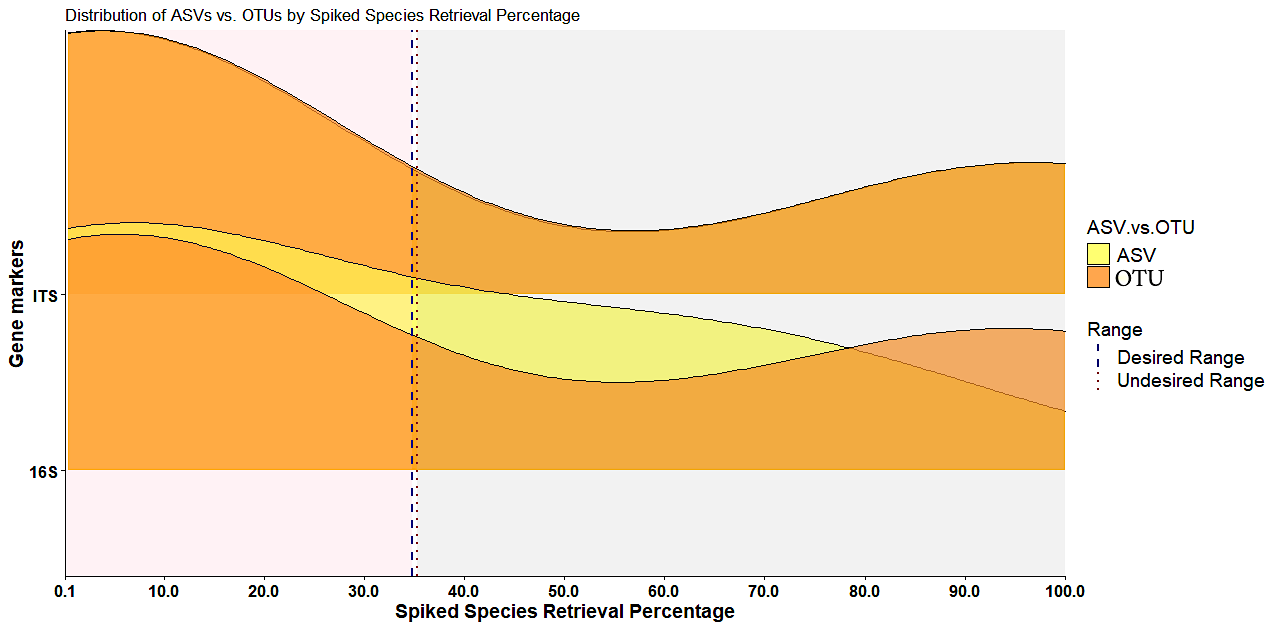

Figure S2 Linear relationships between log₁₀-transformed reads of the bacterial spike-in species (*Tetragenococcus halophilus*) and Hill number q=1 (Hill_q1) across different spike-in recovery windows. Panels represent increasing recovery ranges from 0–10% up to 90–100%, as indicated by color and facet labels.

ITS


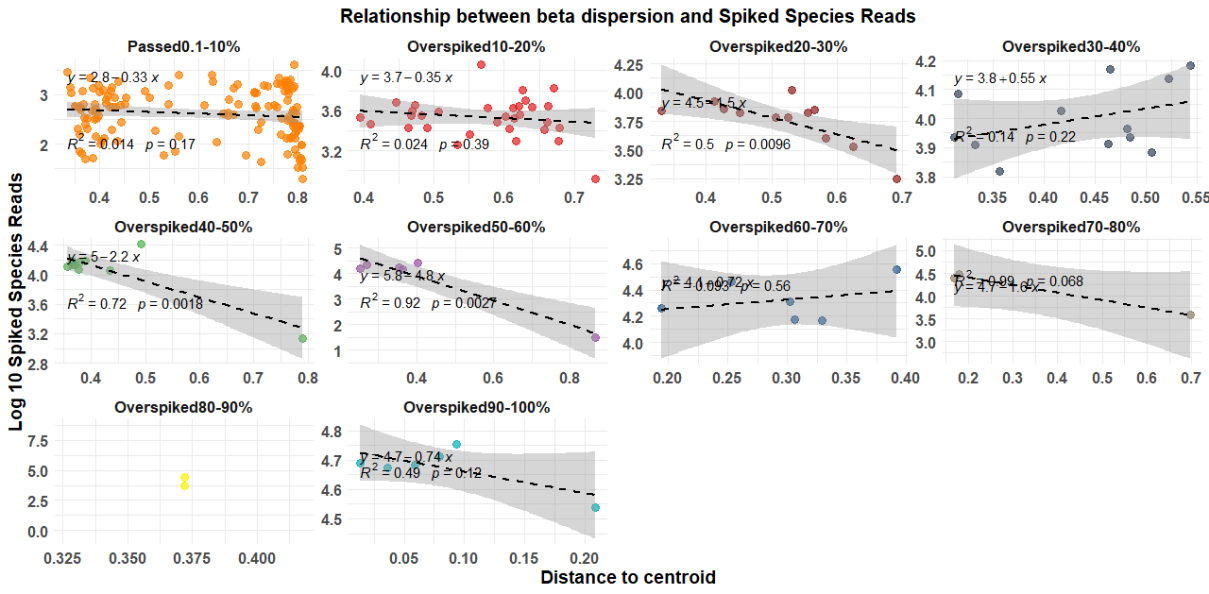

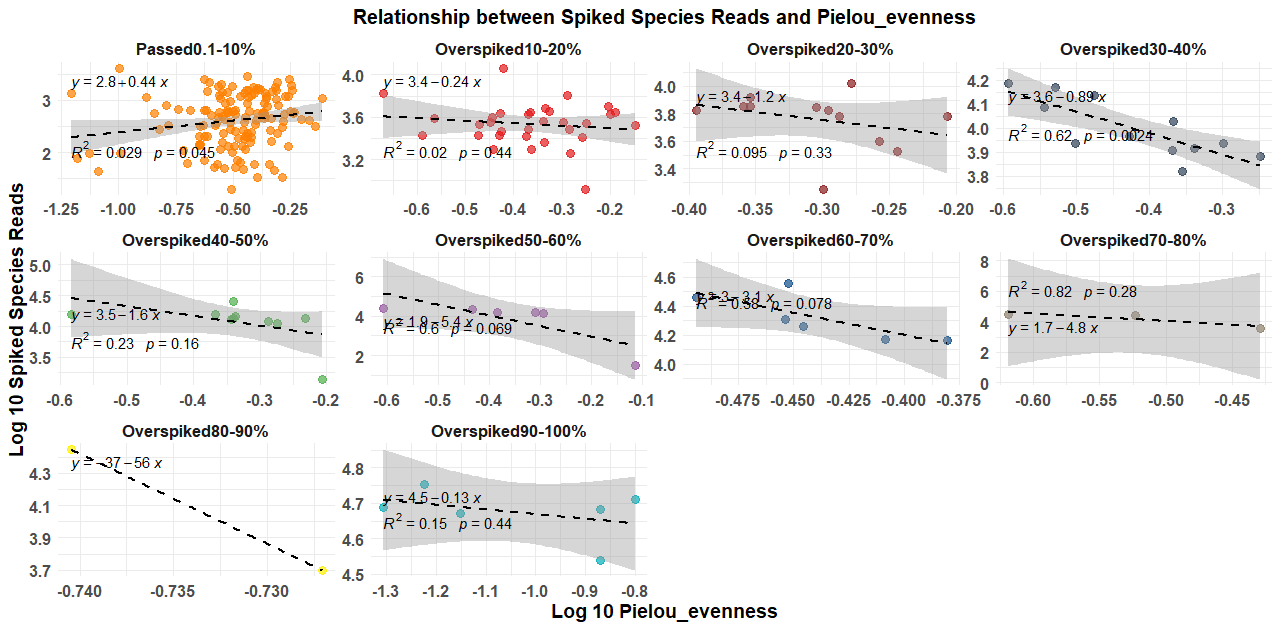

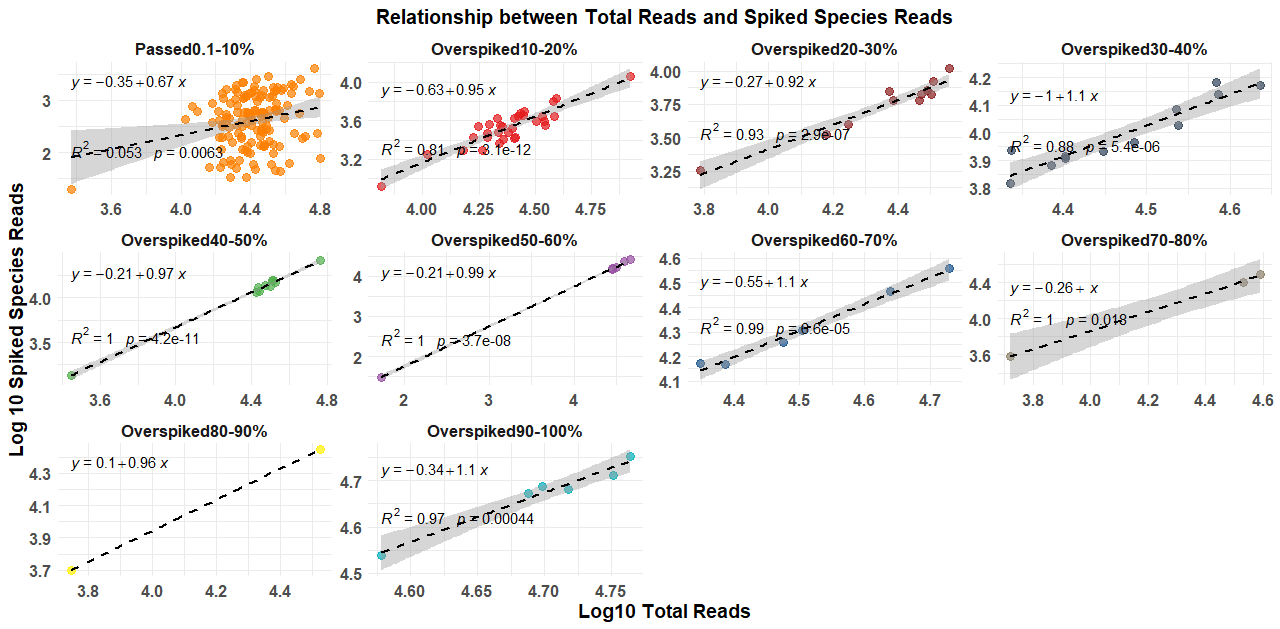

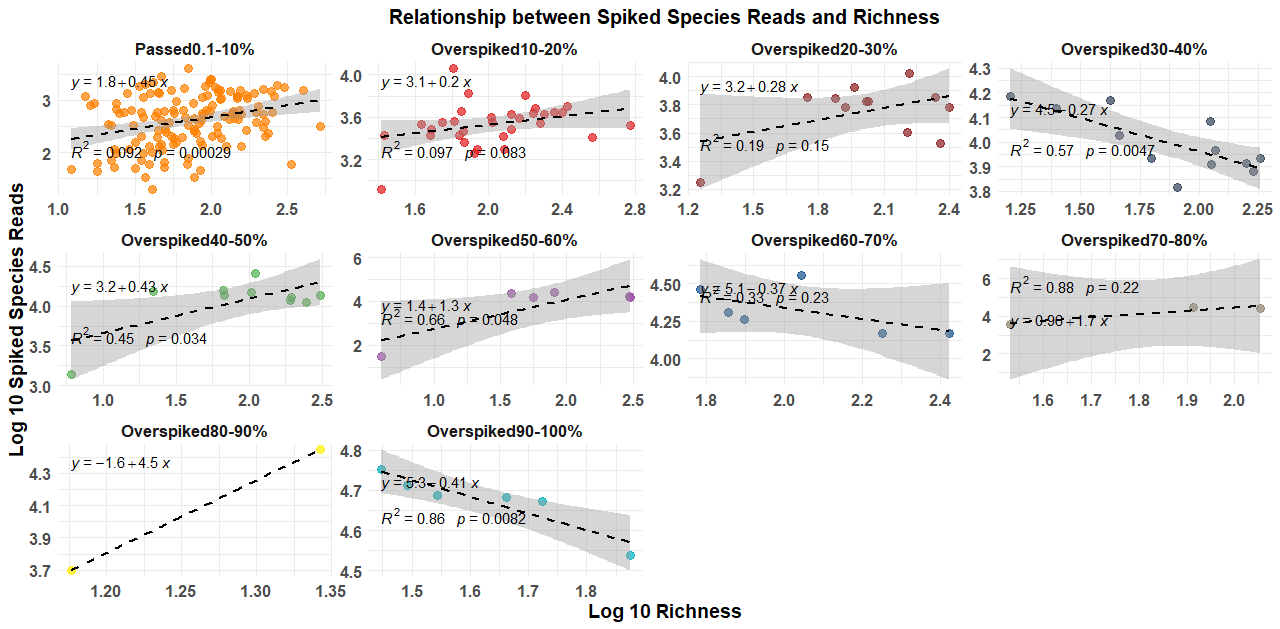


A

B

C

D

Figure S3 shows the linear relationships between spiked species reads and biological indices in the fungal dataset. Panel (A) presents the relationship with richness, while panel (B) depicts the relationship between the abundance of spiked species and the total abundance. Variations in evenness are shown in panel (C), and panel (D) illustrates the variations in distance to centroid with increasing retrieval ranges of spiked species.

Figure S4 Fungal genus distribution across herptile clade orders, shown by absolute abundance (A) and relative abundance (B) in alluvial plots. Plots illustrate the flow of shared bacteria across ecological or life history, including host clade order, ecomode, ecoregion, diet, reproduction type, and metamorphosis status. Similar color-code was used for genus in both plots for easy traceability.


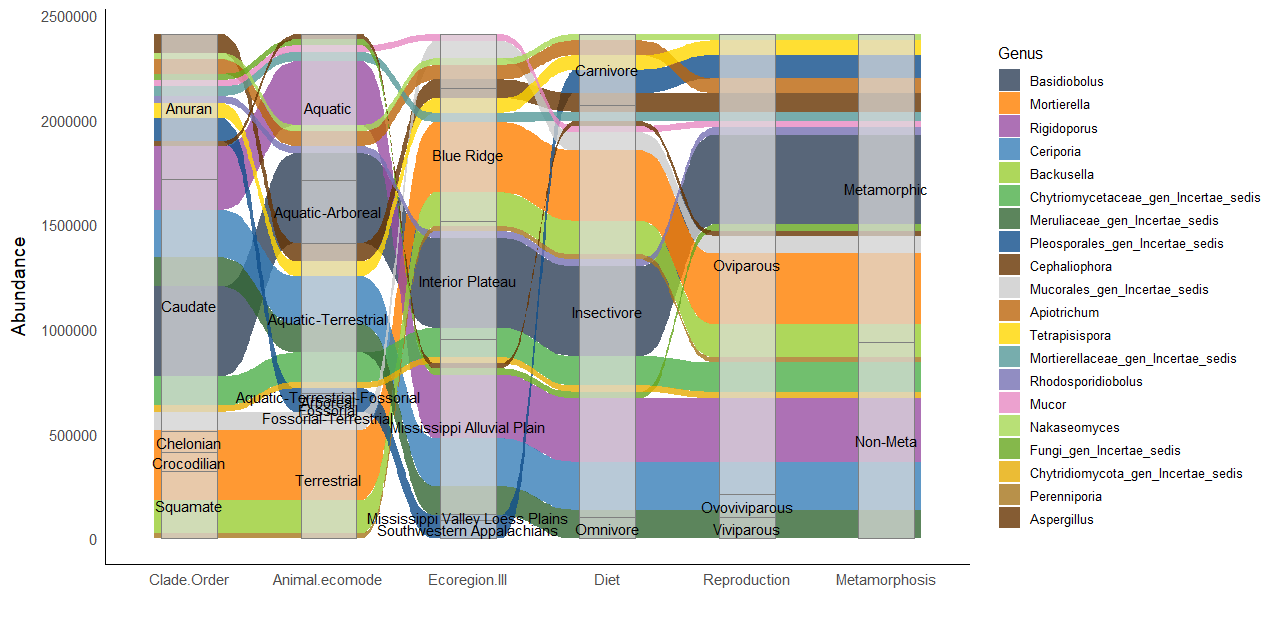

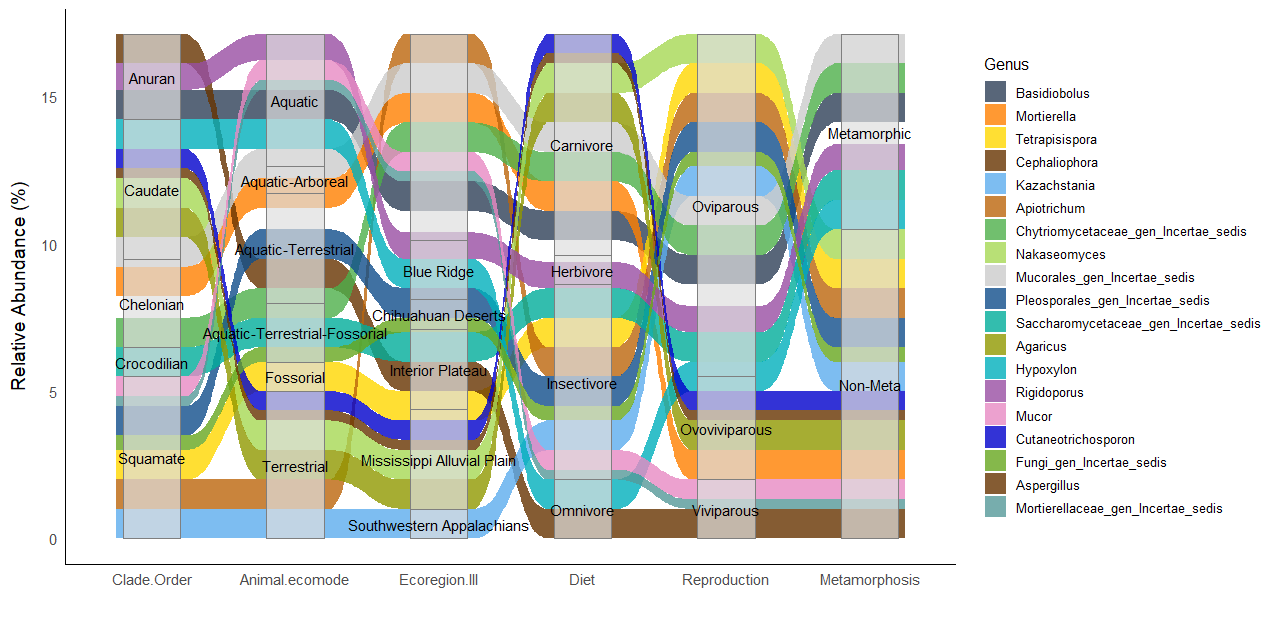


**A**

B


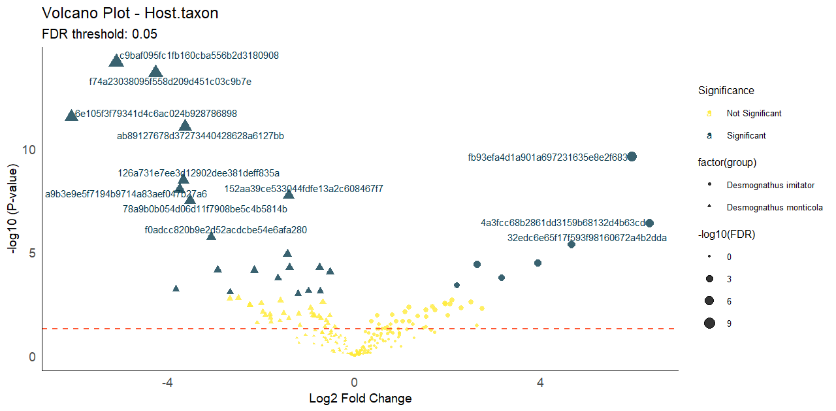

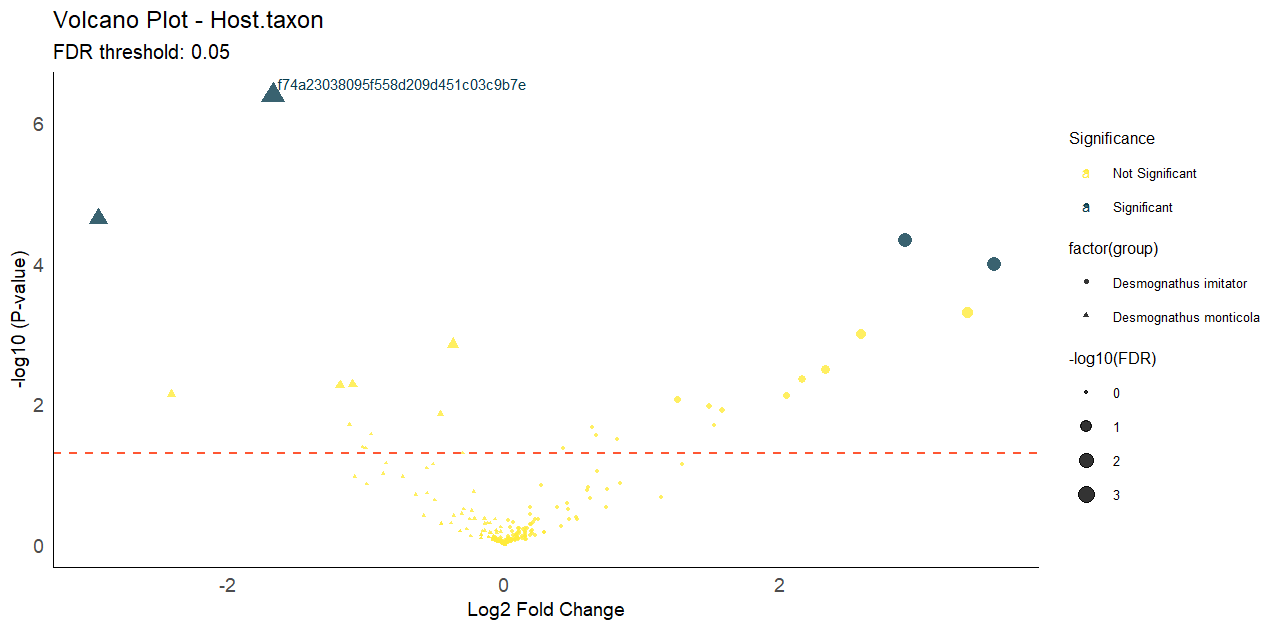

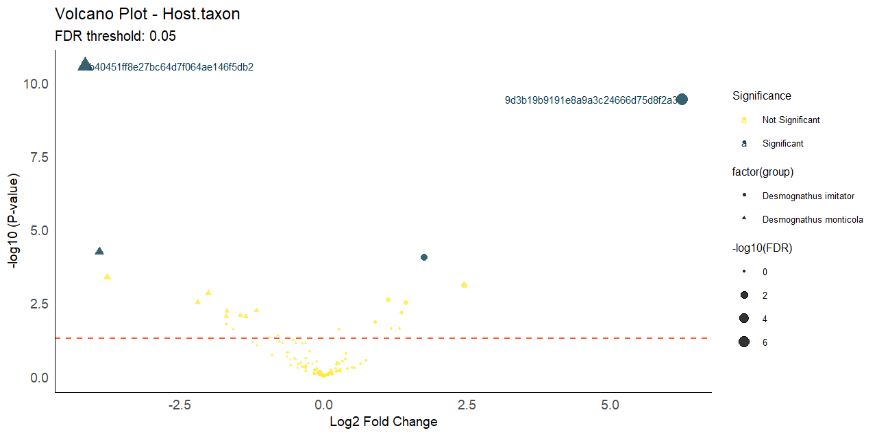

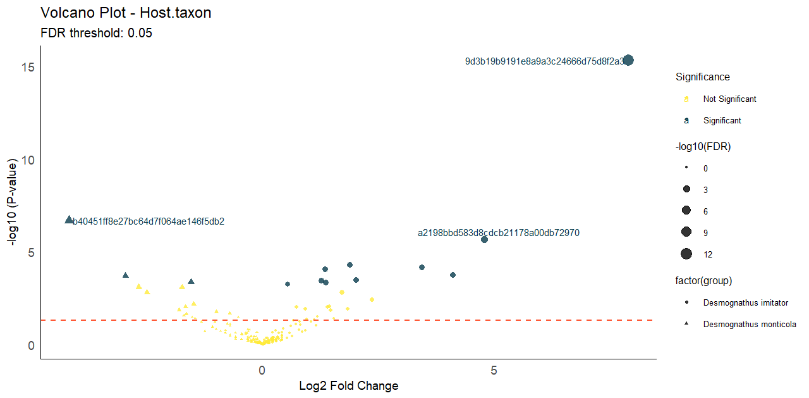


**B**

**Av**

C

D

Figure S5 Volcano plots displaying significant OTUs (green) (padj < 0.05) and nonsignificant OTUs (yellow) (padj > 0.05) for *D. monticola* and *D. imitator*, based on differential abundance analysis using the DEseq2 method in DspikeIn for bacterial absolute (A) and bacterial relative counts (B), fungal absolute (C) and fungal relative counts (D).


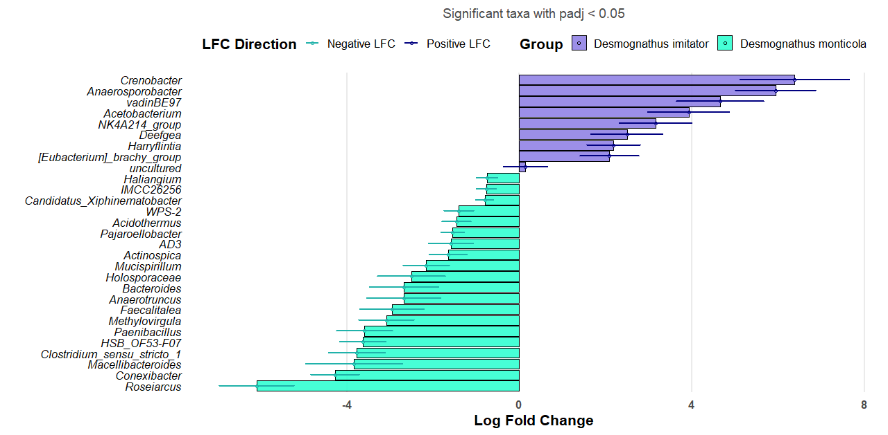

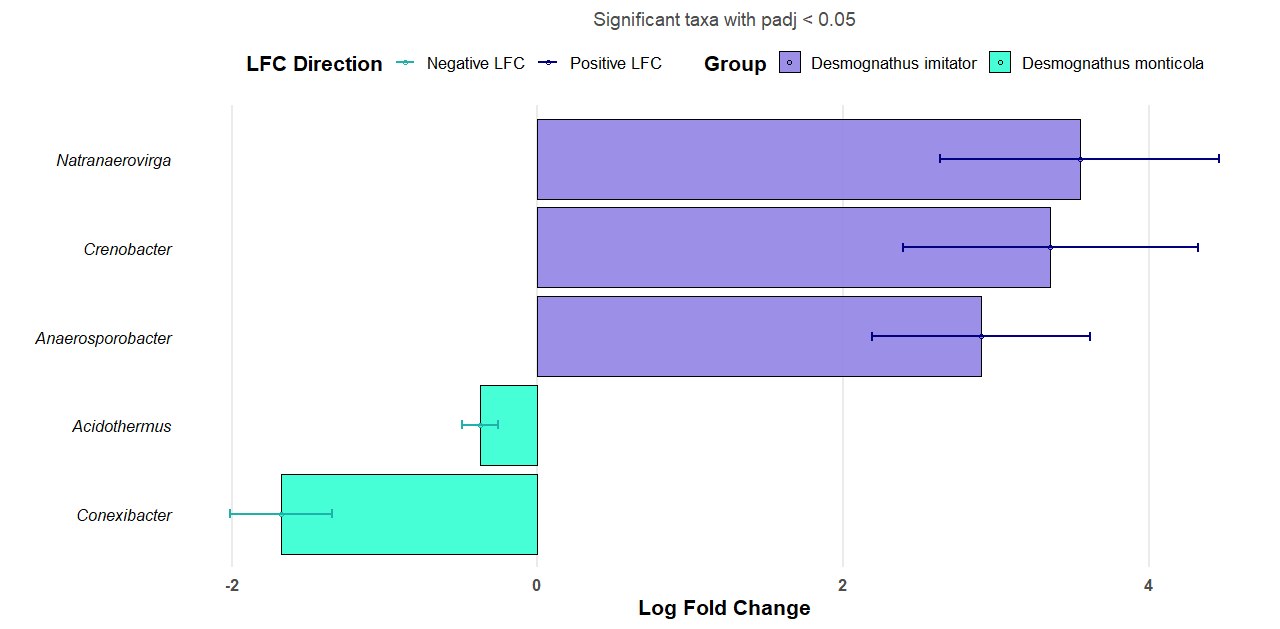

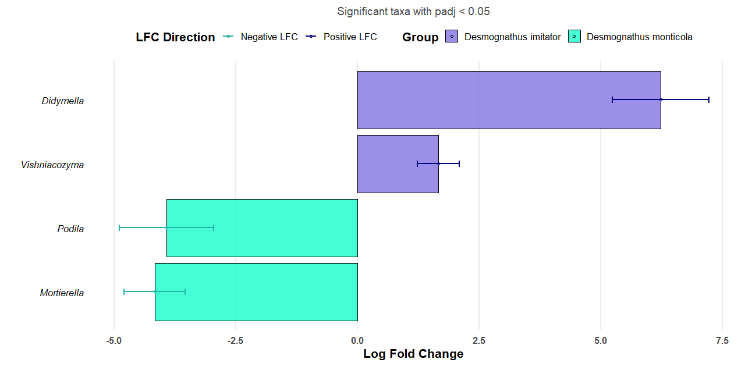

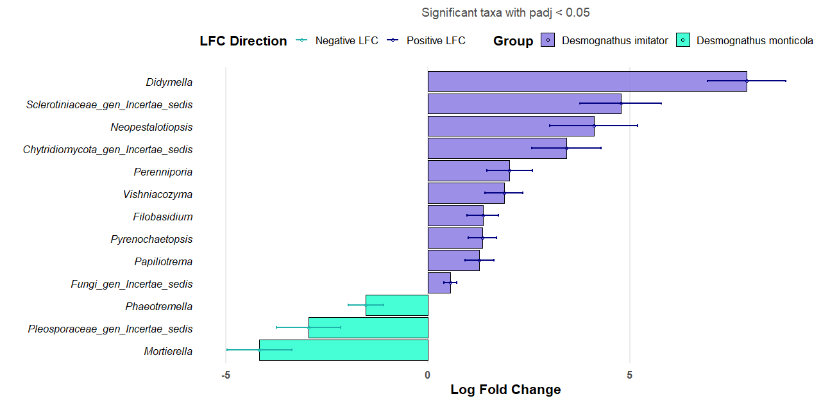


**C**

**D**

**A**

B

Figure S6. Differential abundance analysis using DESeq2 (DspikeIn) identified enriched taxa between *D. monticola* and *D. imitator*. Significantly enriched taxa across hosts are displayed for bacterial absolute abundance (A), bacterial relative abundance (B), fungal absolute abundance (C), and fungal relative abundance (D). Log fold changes (LFC) are also shown for taxa enriched in each host, with negative and positive LFCs indicating enrichment in *D. imitator* and *D. monticola*, respectively.


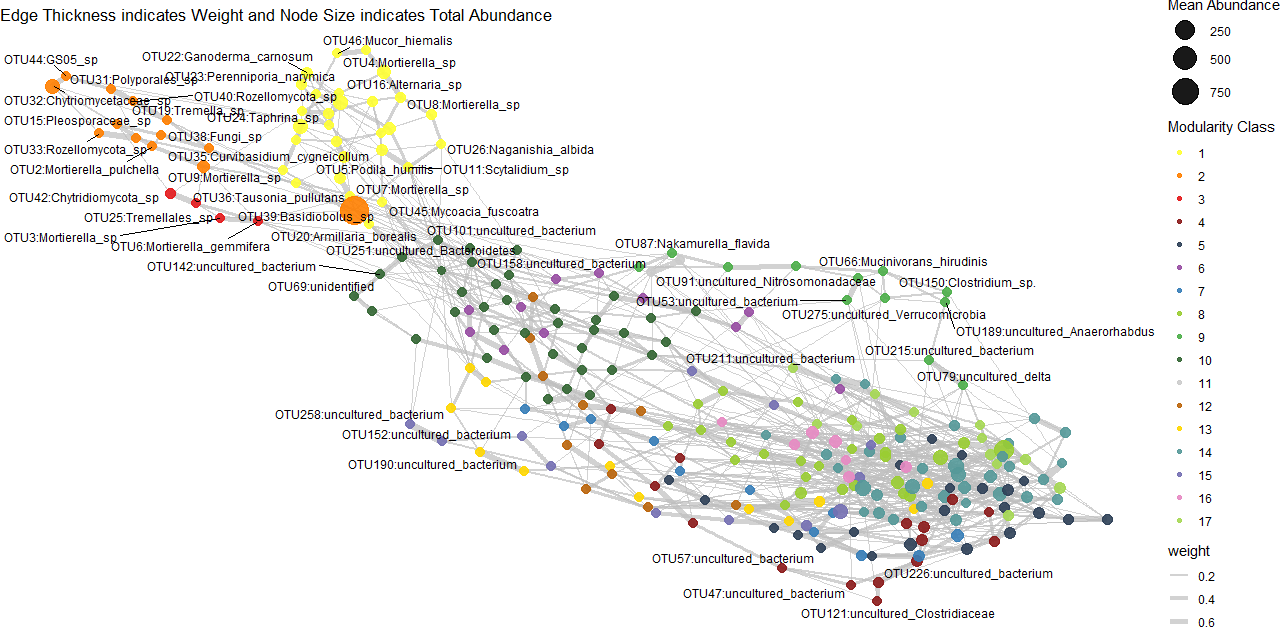

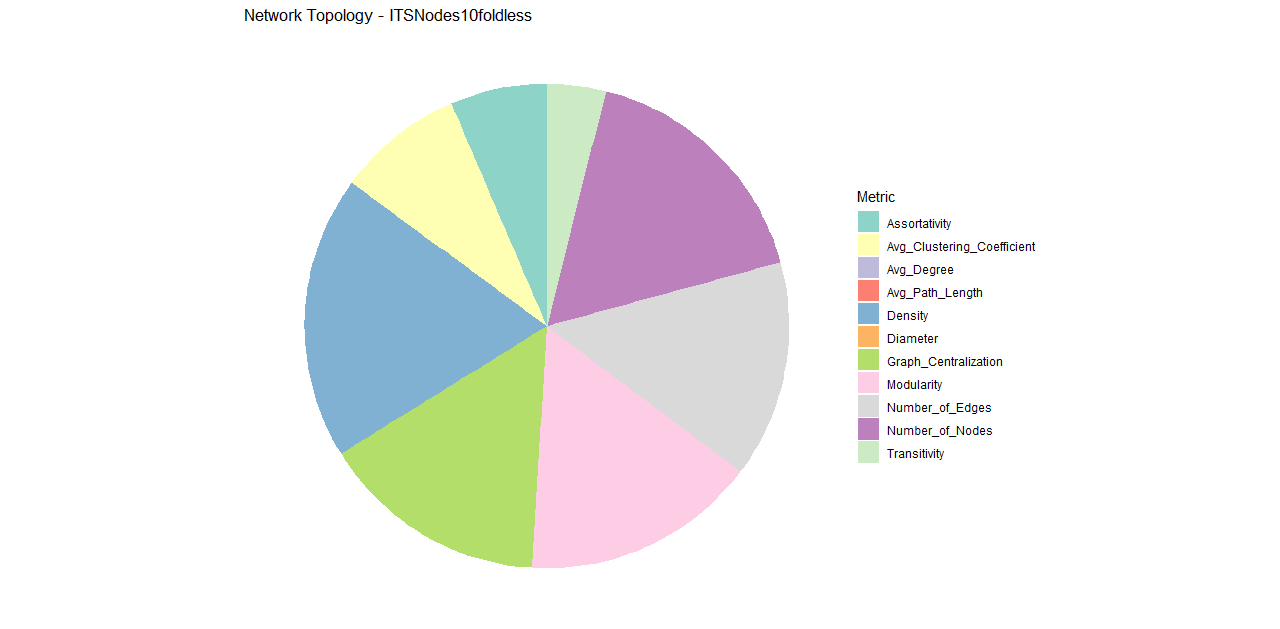

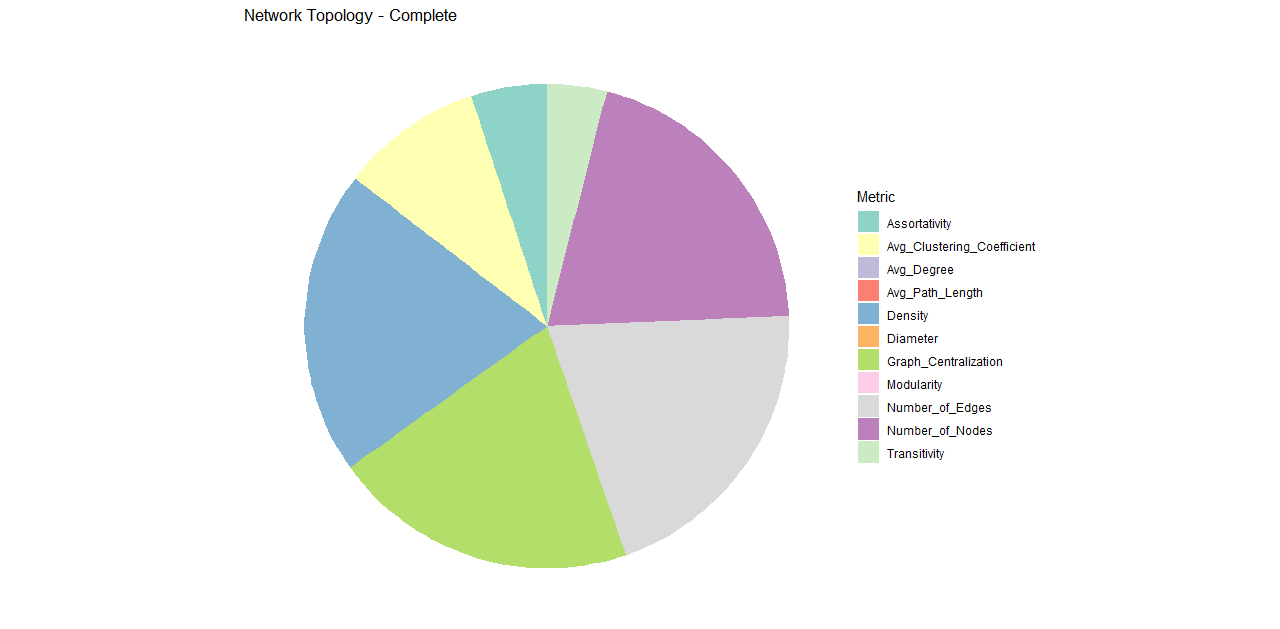


A

C

B

Figure S7 Comparison of network topographic properties across different conditions: (A) complete network, (B) complete network with copy number correction, achieved by dividing all fungal abundances by 10 (C) modularity of cross-domain network associations constructed using absolute counts for the complete network with copy number correction (dividing all fungal abundances by 10) from the gut microbiome of *Dendrobates imitator* and *D. monticola* species. The networks exhibit an organic structure, with edge weight represented by edge thickness, node sizes are representative of mean abundance, and phyla color-coded.


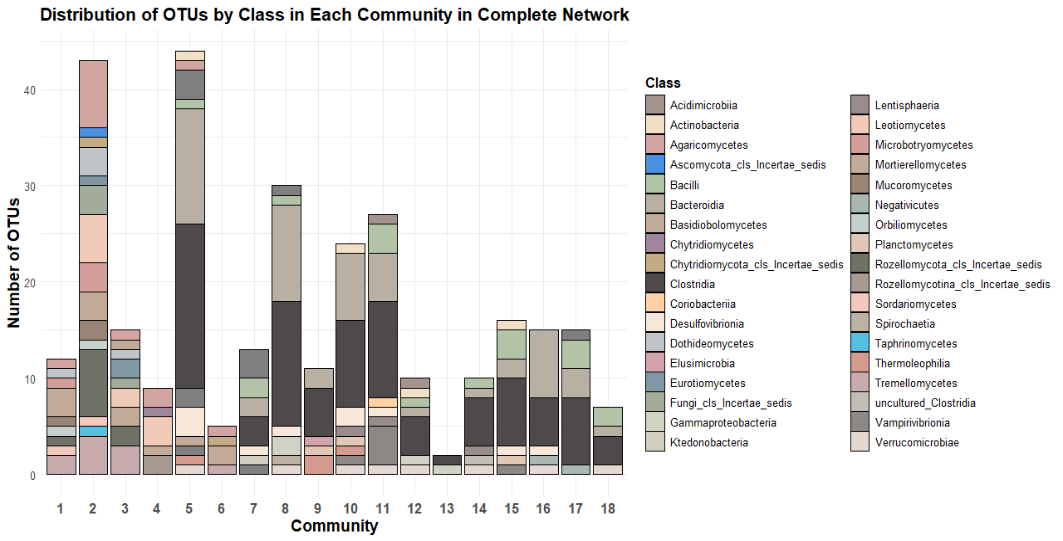

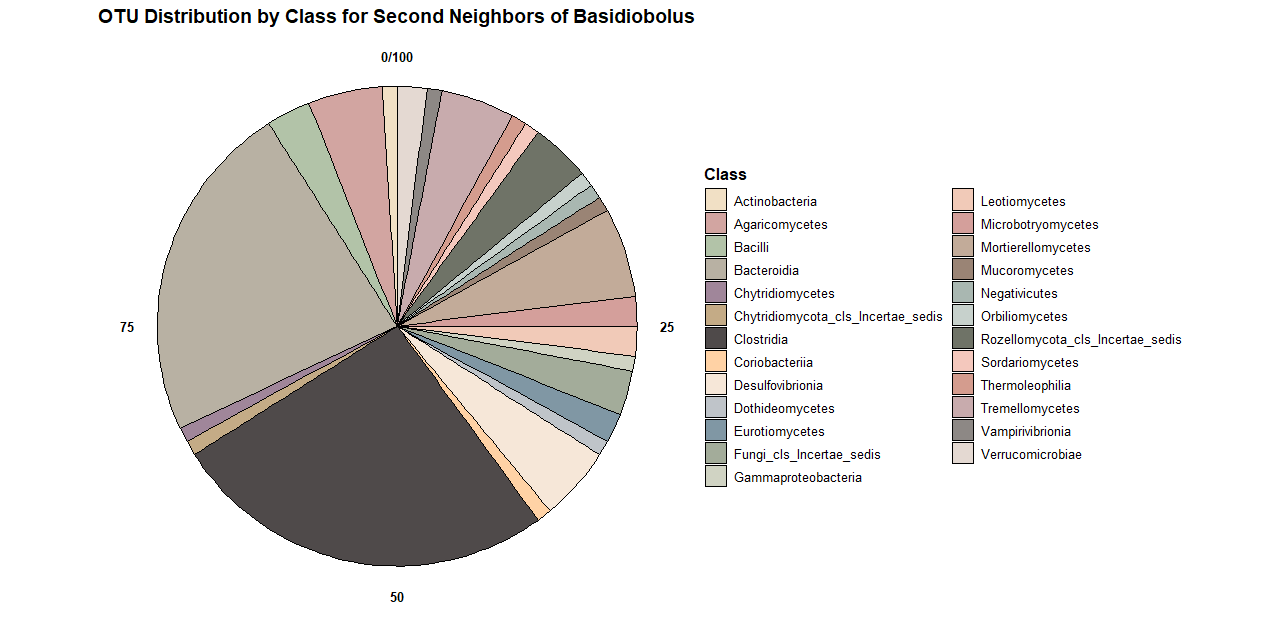

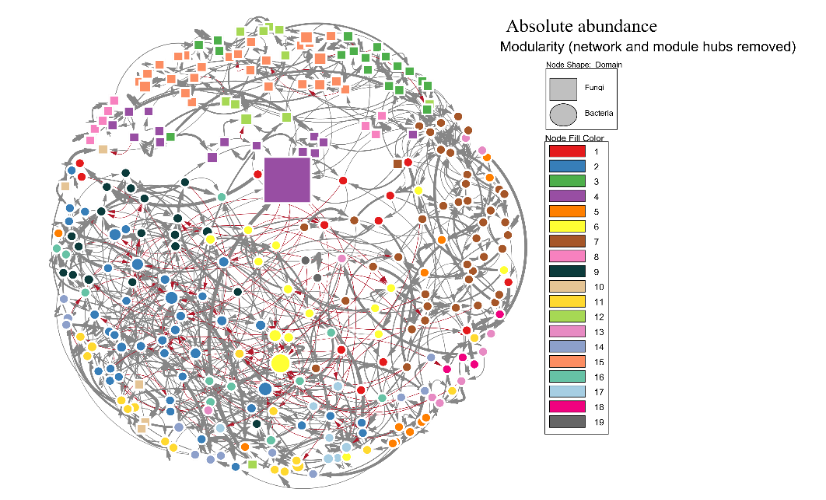

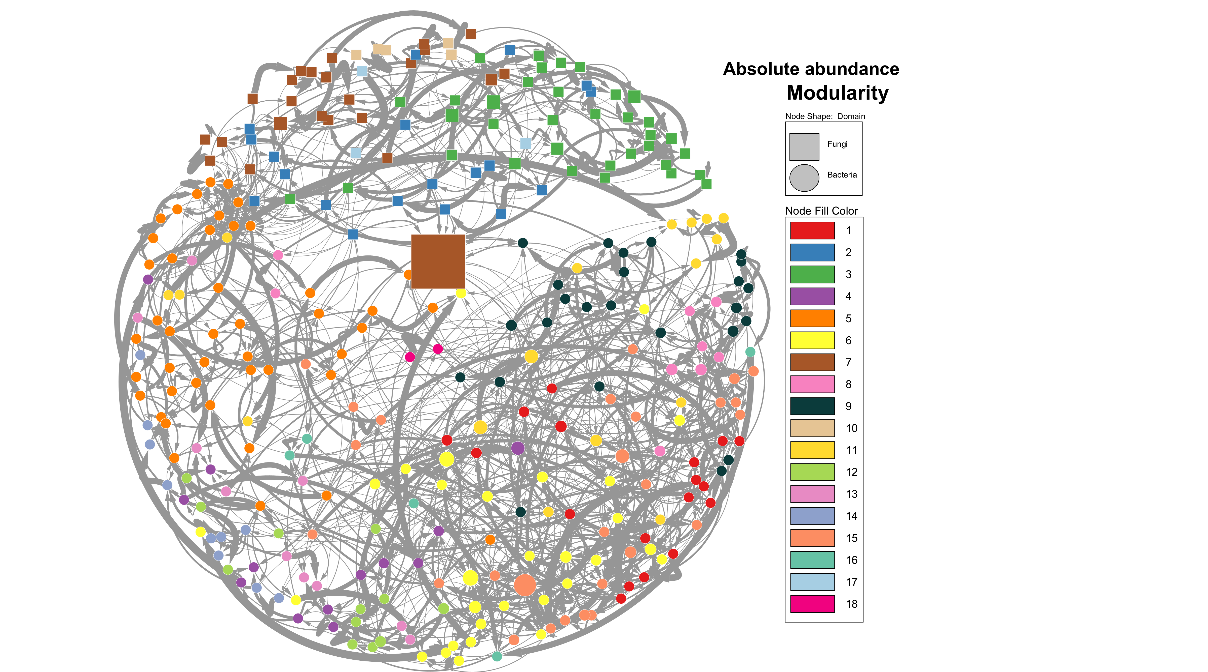

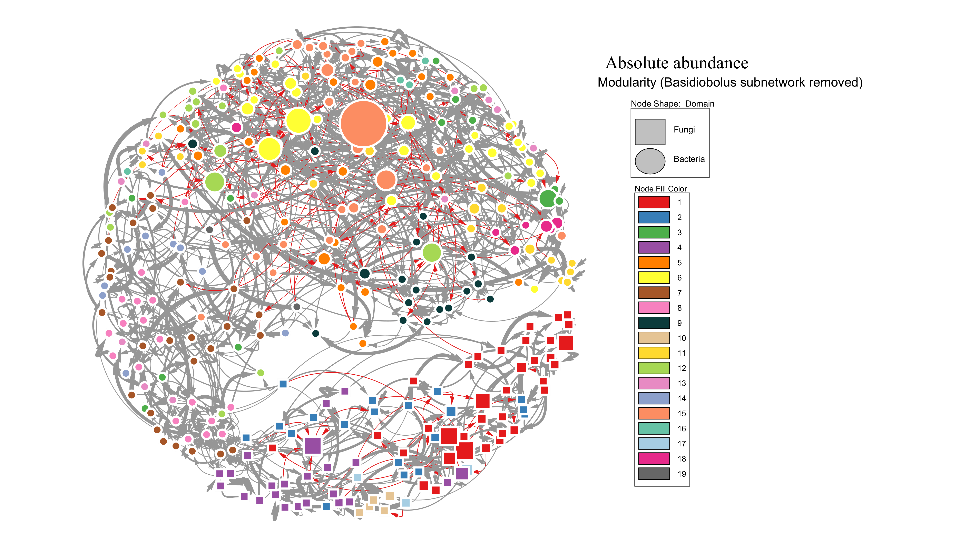


Figure S8 Illustrates the modularity of networks for complete (A), network and module hubs removed (B), and Basidiobolus subnetwork removed (C) from the gut microbiomes of *D. monticola* and *D. imitator*. In these module networks, nodes of the same color represent taxa within the same module. Node size corresponds to the relativized absolute abundance of each taxon, while node shapes distinguish bacteria (circles) from fungi (squares). Edge thickness represents the degree of connections, and edge colors indicate positive (gray) or negative (red) interactions. The module/community members of the complete network are shown in the bar plot (D). Basidiobolus' second neighbors and the number of OTUs for each member are presented in panel (E).

B

A

C

D

E


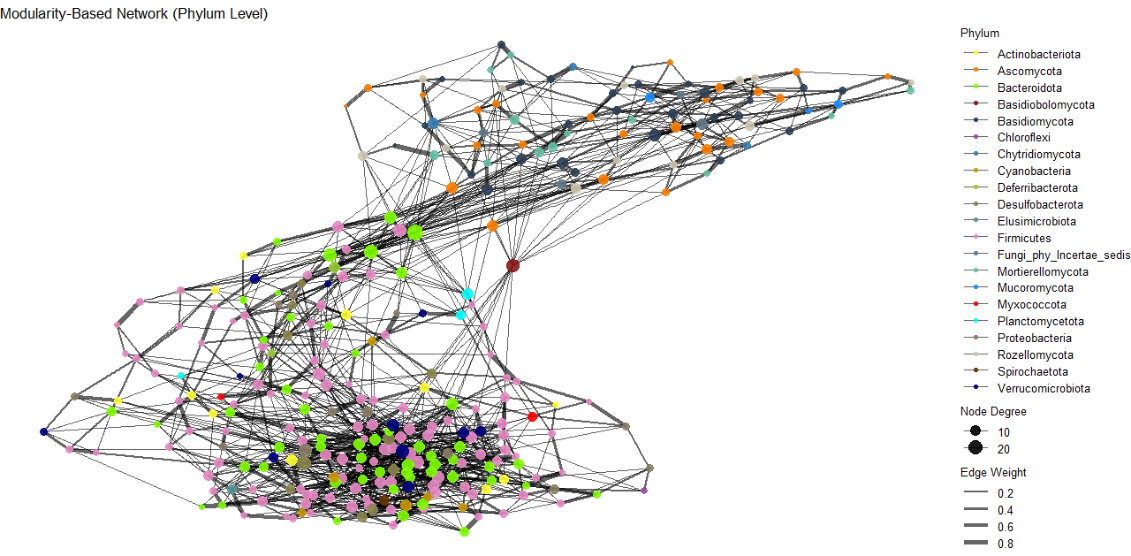

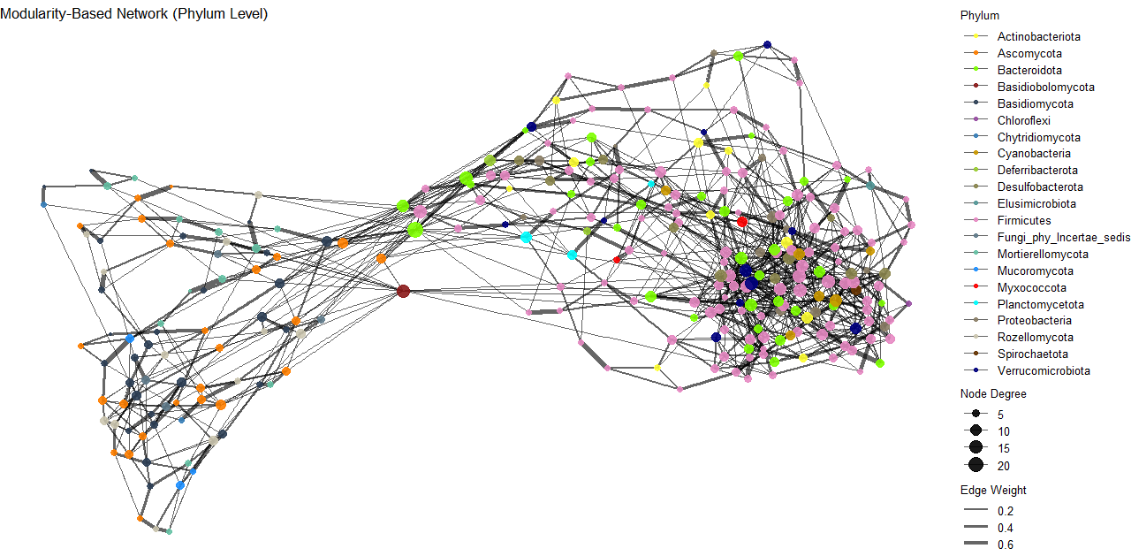

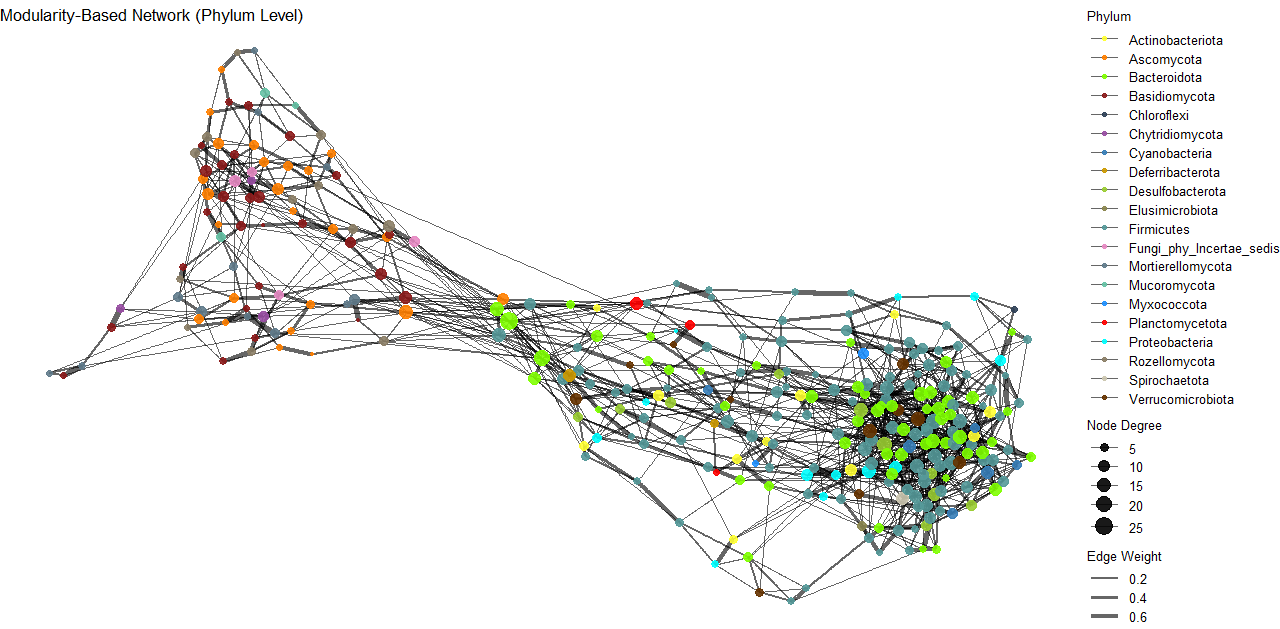


Complete network

C

Network and module Hubs removed

Basidiobolus subnetwork removed

Av

B

Figure S9 shows the modularity of cross-domain network associations constructed using absolute counts for the complete network (A), the network with module hubs removed (B), and the network with the Basidiobolus subnetwork removed (C) from the gut microbiome of *Dendrobates imitator* and *Dendrobates monticola* species. The networks exhibit an organic structure, with edge weight represented by edge thickness, node degree by node size, and phyla color-coded.


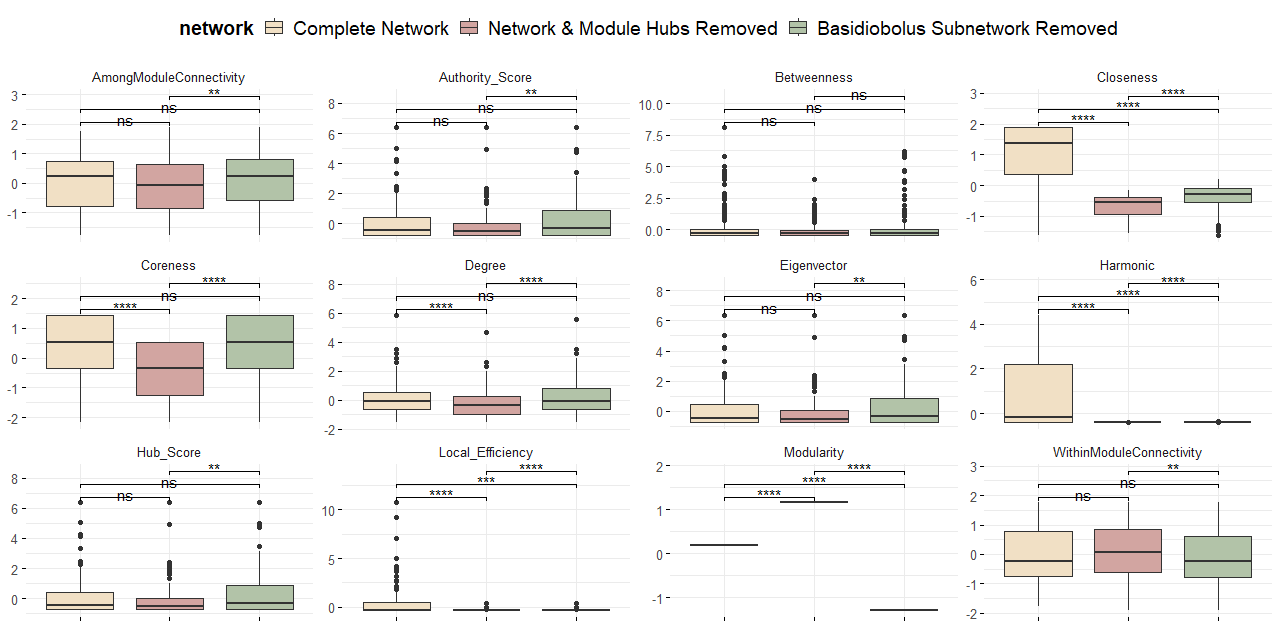


Figure S10 Comparison of network topographic properties across different conditions: (1) complete network, (2) network with module hubs removed, and (3) network with the *Basidiobolus* subnetwork removed


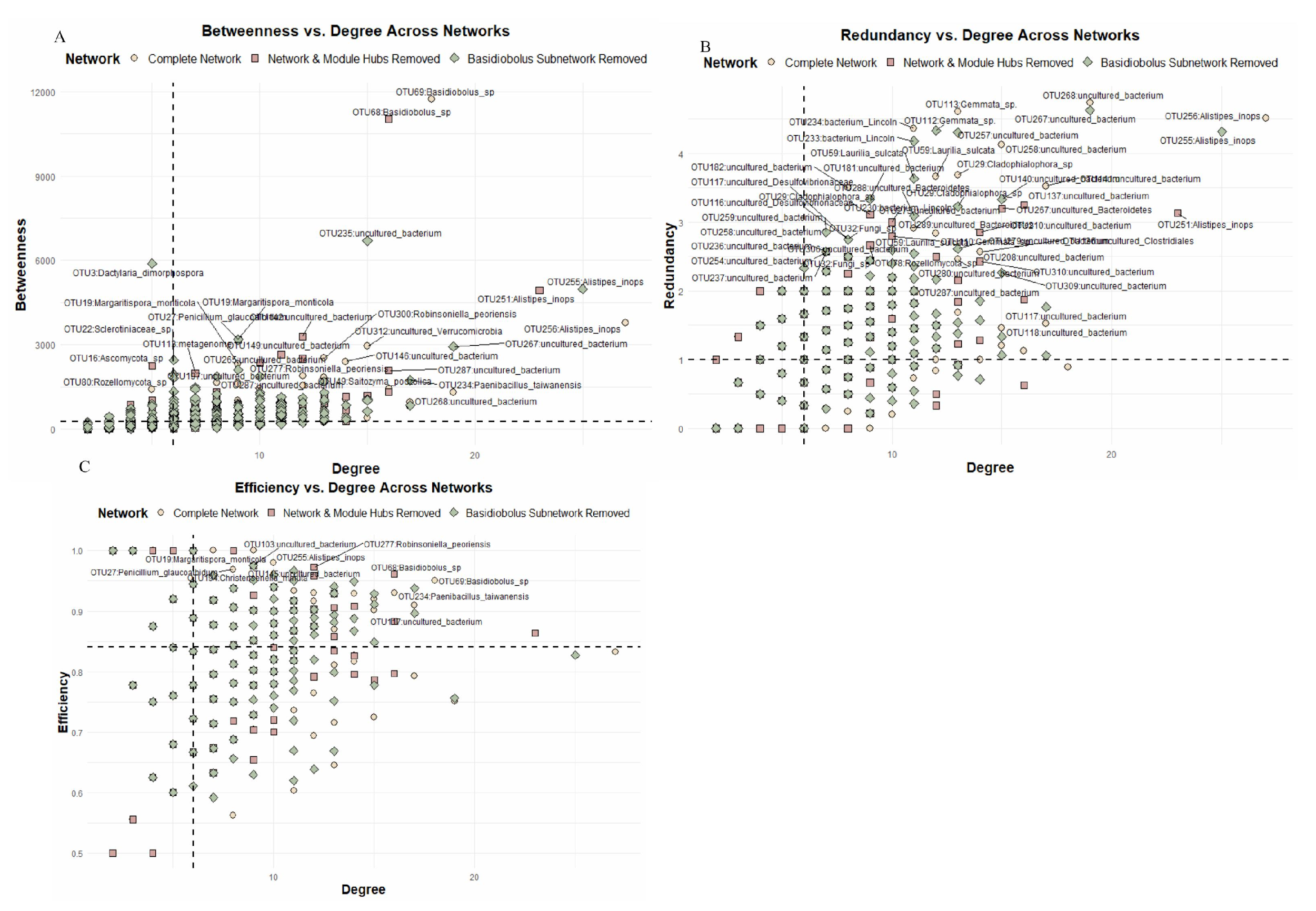


Figure S11 Exploratory analysis mapping structural properties of nodes in gut microbiome networks of *Desmognathus imitator* and *D. monticola*. Plots represent three network configurations: the complete network, a network with module hubs removed, and a network with the *Basidiobolus* subnetwork removed. Metrics include: (A) betweenness, (B) redundancy (number of edges among a node’s neighbors), and (C) efficiency (effective size divided by degree), each plotted against node degree. Dashed lines indicate median values of each axis. These visualizations highlight the diverse topological roles of microbial taxa (such as central, redundant, or bridging nodes) across different network states. Nodes in the top 40% of each metric are labeled for comparative reference. Metric definitions are provided in Table S1.

# DspikeIn Basic functions can be checked using the links and cheatsheet below

<https://rpubs.com/mgh/1294025>

<https://github.com/mghotbi/DspikeIn>

## Vignettes

## Official Vignettes & Documentation

DspikeIn comes with detailed guides and examples to help you get started quickly with both **Phyloseq** and **TreeSummarizedExperiment (TSE)** formats.

### Explore Online

- [**Interactive Guide: DspikeIn with Phyloseq**](https://mghotbi.github.io/DspikeIn/DspikeIn-with-Phyloseq.html)
  Step-by-step usage of DspikeIn with phyloseq objects.
- [**Interactive Guide: DspikeIn with TSE**](https://mghotbi.github.io/DspikeIn/DspikeIn-with-TSE.html)
  Full walkthrough using TreeSummarizedExperiment format.
- [**Documentation Homepage**](https://mghotbi.github.io/DspikeIn/index.html)
  One-click access to all tutorials, stylesheets, and embedded visuals.

### Download for Offline Use

- [Download PDF: DspikeIn with Phyloseq](https://mghotbi.github.io/DspikeIn/DspikeIn-with-Phyloseq.pdf)
- [Download PDF: DspikeIn with TSE](https://mghotbi.github.io/DspikeIn/DspikeIn-with-TSE.pdf)

#
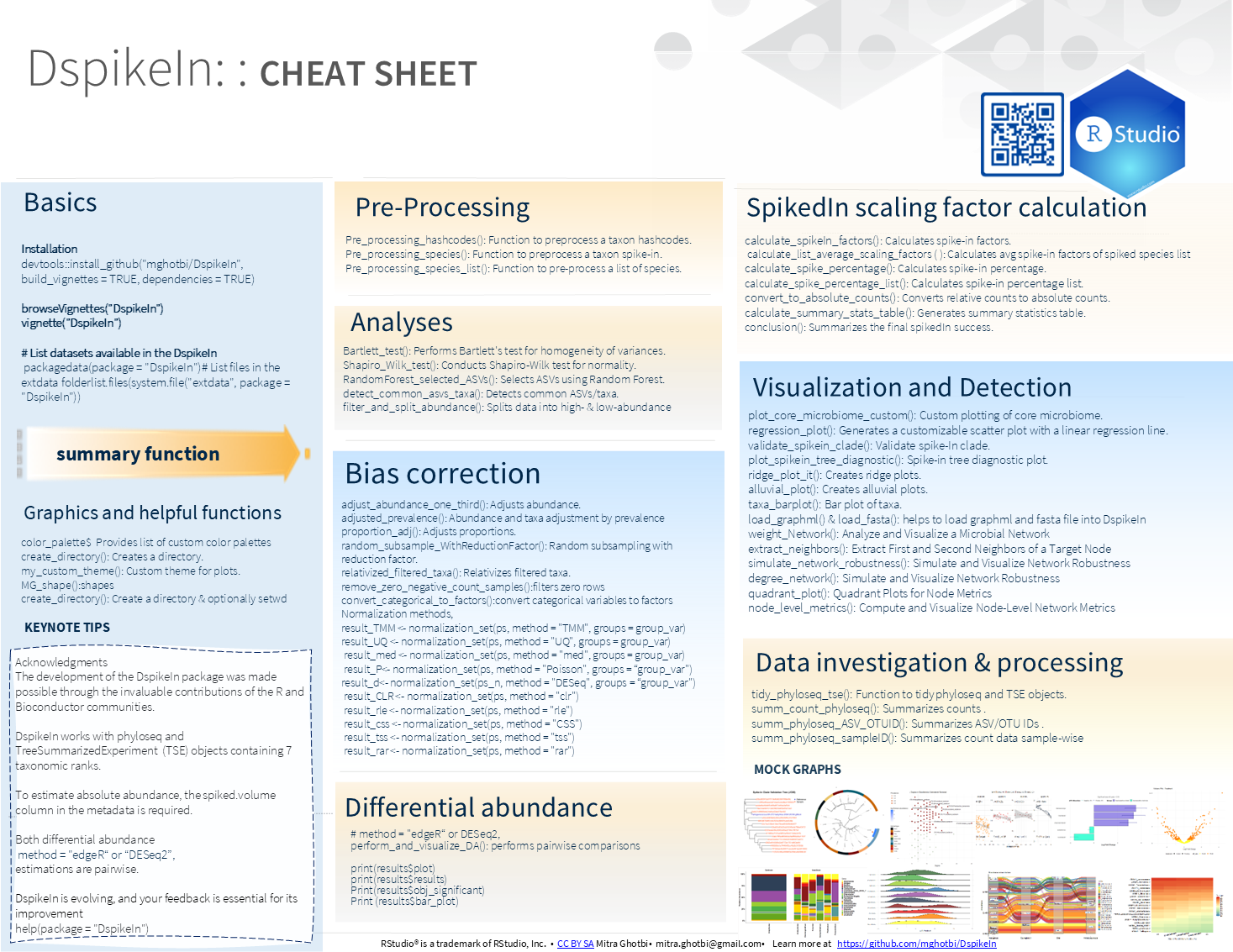
DspikeIn Cheat sheet
